# Alkaline intracellular pH (pHi) increases PI3K activity to promote mTORC1 and mTORC2 signaling and function during growth factor limitation

**DOI:** 10.1016/j.jbc.2023.105097

**Published:** 2023-07-26

**Authors:** Dubek Kazyken, Stephen I. Lentz, Maxwell Wadley, Diane C. Fingar

**Affiliations:** 1 Department of Cell and Developmental Biology, University of Michigan Medical School, Ann Arbor, Michigan, USA; 2 Department of Internal Medicine, Division of Metabolism, Endocrinology, and Diabetes, University of Michigan Medical School, Ann Arbor, Michigan, USA

**Keywords:** pH regulation, target of rapamycin, PI3K, S6 kinase, Akt PKB, cell signaling, protein synthesis, eukaryotic translation initiation factor 4E, eukaryotic translation initiation factor 4E-binding protein, apoptosis

## Abstract

The conserved protein kinase mTOR (mechanistic target of rapamycin) responds to diverse environmental cues to control cell metabolism and promote cell growth, proliferation, and survival as part of two multiprotein complexes, mTOR complex 1 (mTORC1) and mTORC2. Our prior work demonstrated that an alkaline intracellular pH (pHi) increases mTORC2 activity and cell survival in complete media in part by activating AMP-activated protein kinase, a kinase best known to sense energetic stress. It is important to note that an alkaline pHi represents an underappreciated hallmark of cancer cells that promotes their oncogenic behaviors. In addition, mechanisms that control mTORC1 and mTORC2 signaling and function remain incompletely defined, particularly in response to stress conditions. Here, we demonstrate that an alkaline pHi increases phosphatidylinositide 3-kinase (PI3K) activity to promote mTORC1 and mTORC2 signaling in the absence of serum growth factors. Alkaline pHi increases mTORC1 activity through PI3K–Akt signaling, which mediates inhibitory phosphorylation of the upstream proteins tuberous sclerosis complex 2 and proline-rich Akt substrate of 40 kDa and dissociates tuberous sclerosis complex from lysosomal membranes, thus enabling Rheb-mediated activation of mTORC1. Thus, alkaline pHi mimics growth factor–PI3K signaling. Functionally, we also demonstrate that an alkaline pHi increases cap-dependent protein synthesis through inhibitory phosphorylation of eIF4E binding protein 1 and suppresses apoptosis in a PI3K- and mTOR-dependent manner. We speculate that an alkaline pHi promotes a low basal level of cell metabolism (*e.g.*, protein synthesis) that enables cancer cells within growing tumors to proliferate and survive despite limiting growth factors and nutrients, in part through elevated PI3K–mTORC1 and/or PI3K–mTORC2 signaling.

The conserved protein kinase mTOR (mechanistic target of rapamycin) nucleates two multisubunit complexes with distinct substrates and cellular functions, mTORC1 (mTOR complex 1) and mTORC2 (mTOR complex 2) (1, 2, 3, 4, 5, 6). The mutually exclusive mTOR-binding partners Raptor (regulatory-associated protein of mTOR) and Rictor (rapamycin-insensitive companion of mTOR) define mTORC1 or mTORC2, respectively (7, 8, 9, 10). In mammals, these mTOR complexes sense and respond to hormones (*e.g.*, insulin), growth factors (*e.g.*, IGF, insulin-like growth factor), metabolites (*e.g.*, amino acids, glucose, and energy/ATP), and stress to control cell metabolism, growth, proliferation, and survival (4, 5, 6). As such, their dysregulation contributes to pathological states, such as cancer, diabetes, and neurodegenerative disorders (1, 4).

Insulin and IGF activate mTORC1 through the phosphatidylinositide 3-kinase (PI3K)–Akt pathway (11, 12). Receptor-mediated activation of PI3K generates PIP_3_ (PI-3,4,5-P3), which enables PDK1 (3-phosphoinositide-dependent protein kinase-1) to phosphorylate and activate Akt through phosphorylation of T308, the essential activation loop site. Akt in turn phosphorylates tuberous sclerosis complex 2 (TSC2) to inhibit TSC function by inducing its dissociation from lysosomal membranes, where the mTORC1-activating Rheb GTPase resides (13, 14, 15). As TSC2 functions as a GAP for Rheb, dissociation of TSC from lysosomes increases Rheb GTP loading through a spatial control mechanism (16, 17, 18). Rheb-GTP in turn binds to and activates mTORC1 directly through global conformational changes in mTORC1 structure (19, 20, 21). Well-studied substrates of mTORC1 include the ribosomal S6 protein kinase 1 (S6K1) (on P-T389) and eIF4E binding protein 1 (4EBP1) (on multiple sites), which promote cap-dependent protein synthesis, and ULK1 (on P-S757), which suppresses autophagy during amino acid sufficiency (6). The regulation of mTORC2 remains less well defined (3, 5). The insulin–IGF pathway activates mTORC2 in a PI3K-dependent manner. Direct binding of the PI3K product PIP_3_ to the pH domain of mSin1, an exclusive mTORC2 partner protein, was shown to attenuate autoinhibition of the mTORC2 kinase domain through conformational changes (22). A well-studied substrate of mTORC2 includes Akt (PKB) (23). While not essential for Akt activity, mTORC2-mediated phosphorylation of Akt on its hydrophobic motif site (S473) boosts Akt activity and modifies substrate preference (24, 25, 26).

Activation of mTORC1 but not mTORC2 by insulin–IGF requires sufficient levels of cellular amino acids (2, 4). As such, mTORC1 functions as an amino acid sensor that fine-tunes metabolic activity in accordance with environmental cues (2, 4). mTORC1 senses intracellular amino acids through several multisubunit complexes (*e.g.*, the GATOR2, GATOR1, and Ragulator–LAMTOR complexes), which converge on heterodimeric Rag GTPases (Rag A or B bound to Rag C or D) tethered to lysosomal membranes. Amino acid stimulation of amino acid–starved cells increases GTP loading of RagA or RagB, which recruits mTORC1 to lysosomal membranes through its Raptor subunit (27, 28). In simple terms, the insulin–PI3K–Akt pathway and the amino acid–Rag pathway signal in parallel to activate mTORC1 on lysosomal membranes. Emerging data indicate greater complexity in how growth factors and amino acids cooperate to regulate mTORC1 appropriately, however. For example, TSC localizes to lysosomes in amino acid–starved cells cultured with serum growth factors (*i.e.*, fetal bovine serum [FBS]), and the readdition of amino acids induces the rapid dissociation of TSC from lysosomal membranes (29, 30). Thus, TSC only fully dissociates from lysosomes in cells cultured with sufficient levels of growth factors and amino acids (29, 30). In addition, growth factor signaling increases the mTORC1-mediated phosphorylation of RagC, which promotes mTORC1 signaling in an autoregulatory manner (31), and amino acids increase polyubiquitination of Rheb, which facilitates the mTORC1–Rheb interaction (32). Thus, crosstalk occurs between growth factor and amino acid signaling pathways. Various types of cell stress including hyperosmotic stress, energetic stress, hypoxia, and high pH (*i.e.*, pH 9.4) also recruit TSC to lysosomes to inhibit mTORC1 (30). Thus, growth factor, amino acid, and cell stress signaling converge on TSC to control mTORC1 activity through Rheb (29, 30). Taken together, these findings indicate that complex pathways and mechanisms work together to fine-tune mTORC1 activity.

Cap-dependent protein synthesis represents a fundamental cellular process promoted by mTORC1 (33, 34). mTORC1 mediates the sequential phosphorylation of the translational inhibitor 4EBP1 on multiple sites (first at T37 and T46 and subsequently at T70 and S65) (33, 34, 35). In the hypophosphorylated state, 4EBP1 binds to and inhibits eIF4E (eukaryotic initiation factor 4E), a rate-limiting translation initiation factor that binds to the cap structure on the 5′ ends of mRNA transcripts. mTORC1-mediated phosphorylation of 4EBP1 causes its dissociation from eIF4E, enabling the binding of eIF4G and the recruitment of other translation initiation factors to assemble the eIF4F translation preinitiation complex (33, 34). mTORC1 signaling to S6K1 also promotes protein synthesis by upregulating rRNA transcription (aka, ribosome biogenesis) and through the phosphorylation of translation factors (*e.g.*, eIF4B, PDCD4) and likely other translation-related factors (*e.g.*, the ribosomal protein S6) (33, 34).

Cell survival and metabolism represent fundamental cellular processes controlled by mTORC2 (5, 11, 12). Our prior work demonstrated that AMPK (AMP-activated protein kinase), a kinase best known to sense and respond to low levels of cellular energy (*i.e.*, elevated AMP or ADP), phosphorylates mTOR and Rictor to activate mTORC2 directly in response to energetic stress, which suppresses apoptosis (36). This work not only identified energetic stress as a previously unknown activator of mTORC2 but provided a molecular explanation for the AMPK-cancer conundrum in which AMPK functions as either a tumor suppressor (at early stages of oncogenesis through suppression of mTORC1) or a tumor promoter (at later stages of oncogenesis through poorly defined mechanisms) (36, 37, 38, 39, 40). We proposed that the AMPK–mTORC2 axis may promote tumorigenesis by increasing the survival of cancer cells experiencing energetic stress within the core of growing tumors starved of essential factors (*e.g.*, growth factors, nutrients, oxygen). Our subsequent work demonstrated that another type of cell stress, that is, elevated intracellular pH (pHi), increases AMPK activity to promote mTORC2 signaling in cooperation with an unknown input to suppress apoptosis during growth factor limitation (41). Interestingly, tumor tissue often displays a reversal of the pH gradient whereby cancer cells possess a slightly alkaline pHi within an acidic extracellular space, the reverse of normal tissue (42, 43, 44, 45). Moreover, we now appreciate that elevated pHi promotes diverse cancer cell behaviors, including metabolic adaptation, cell proliferation, evasion from apoptosis, and cell migration (42, 43, 44, 45, 46).

Here, we identify pHi as a previously unknown regulator of PI3K. We found that alkaline pHi increases PI3K activity, which promotes PI3K-dependent mTORC1 and mTORC2 signaling, cap-dependent translation, and cell survival during growth factor limitation. Thus, alkaline pHi mimics the growth factor–PI3K pathway. This study also identifies PI3K as the potential elusive input that increases mTORC2 signaling in response to alkaline pHi in cooperation with AMPK from our previous work (41). Taken together, these results suggest that elevated PI3K–mTORC1 and/or PI3K–mTORC2 signaling may drive oncogenic cell behaviors mediated by increased pHi in cancer cells, possibly by promoting the growth and survival of cancer cells within growing tumors deprived of essential growth factors.

## Results

### Alkaline extracellular pH (pHe) increases mTORC1 and mTORC2 signaling in the absence of serum growth factors

**Figure 1:**
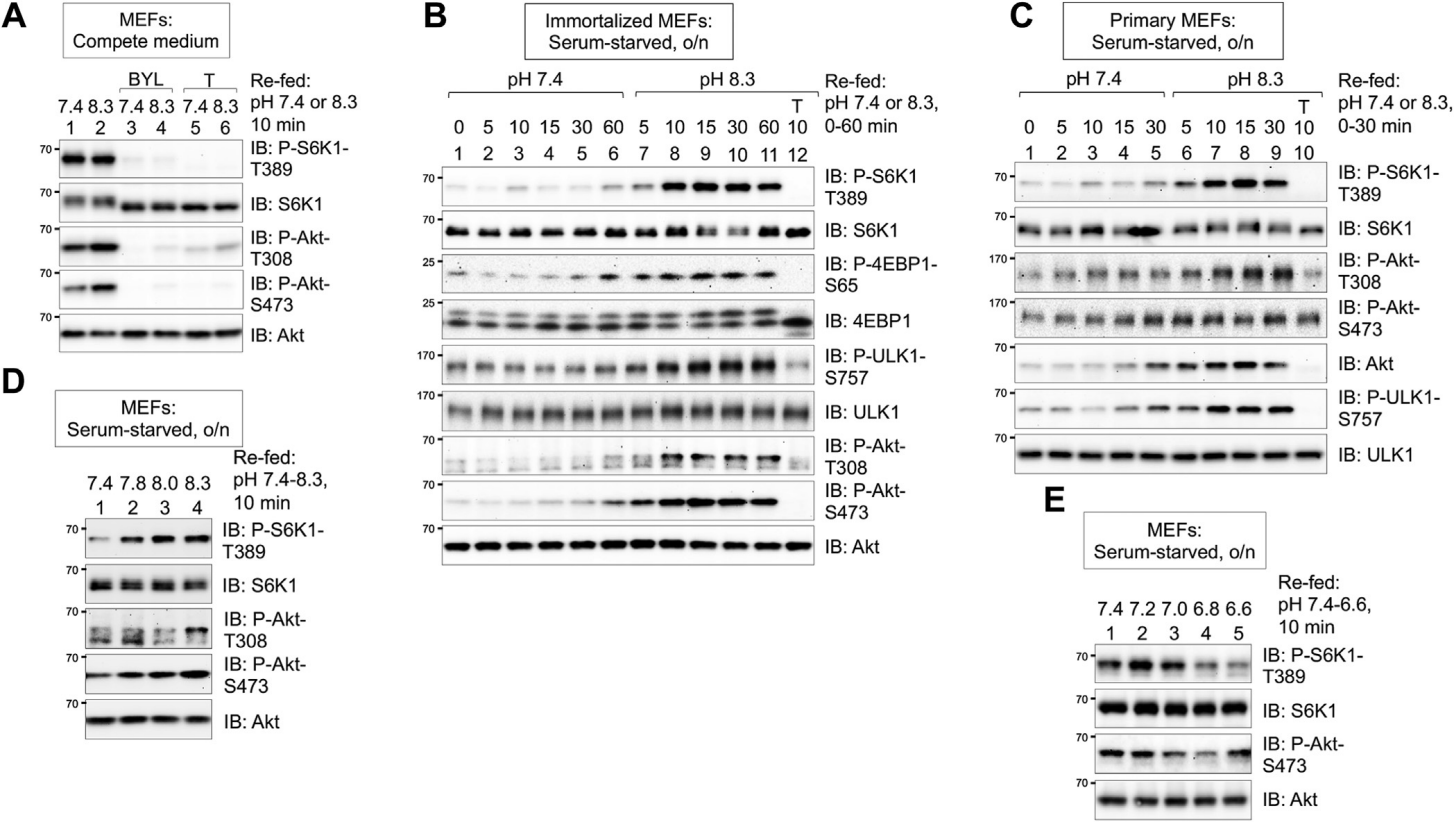
**Alkaline extracellular pH (pHe) increases mTORC1 and mTORC2 signaling in the absence of serum growth factors.***A*, MEFs were pretreated without or with BYL719 (BYL) (10 μM) or Torin1 (T) (100 nM) (30 min) and refed with complete DMEM at pH 7.4 or pH 8.3 (10 min) in the absence or presence of drugs. Whole-cell lysates were immunoblotted as indicated. *B*, immortalized MEFs were serum-starved overnight (o/n), pretreated without or with Torin1 (T) (100 nM) (30 min), and refed with serum-free DMEM at pH 7.4 or pH 8.3 (0–60 min) in the absence or the presence of Torin1. Whole-cell lysates were immunoblotted as indicated. ∗Note: From here on, “MEFs” refer to immortalized MEFs. *C*, primary MEFs were treated as in *B*. *D*, MEFs were serum-starved o/n and refed with serum-free DMEM at pH 7.4, 7.8, 8.0, or 8.3 (10 min). *E*, MEFs were serum-starved o/n and refed with serum-free DMEM at pH 7.4, 7.2, 7.0, 6.8, or 6.6 (10 min). All panels show representative results from at least three independent experiments. Molecular weight markers are provided in kilodalton. DMEM, Dulbecco’s modified Eagle’s medium; MEF, mouse embryonic fibroblast; mTORC1, mTOR complex 1.

While investigating the activation of the AMPK–mTORC2 axis by alkaline pHi in our prior work (41), we noted that refeeding mouse embryonic fibroblasts (MEFs) cultured in complete media (CM; *i.e.*, Dulbecco's modified Eagle's medium [DMEM] containing FBS and amino acids) at an alkaline pH 8.3 increased the phosphorylation of Akt S473 but not S6K1 T389 in a manner sensitive to Torin1 (an ATP-competitive mTOR inhibitor) (41) (Fig. 1*A*). Thus, an alkaline pHe is sufficient to increase mTORC2 but not mTORC1 signaling in the presence of growth factors. It is important to note that Demetriades *et al.* (30) also found that raising the pH of CM (*i.e.*, to pH 8.4) had no effect on mTORC1 signaling to S6K1 in MEFs. To better understand this finding, we found that inhibition of class I PI3Kα with the drug BYL719 (aka, alpelisib) blocked the increase in Akt P-S473 mediated by alkaline pHe in CM (Fig. 1*A*). As growth factors represent a strong activating input to mTORC1, we reasoned that we should analyze serum-starved MEFs in order to probe a potential role for alkaline pH in the regulation of mTORC1. Refeeding serum-starved immortalized (Fig. 1*B*) or primary MEFs (Fig. 1*C*) with serum-free DMEM at pH 8.3 for 5 to 60 min increased the phosphorylation of several mTORC1 substrates, that is, S6K1-T389, 4EBP1-S65, and ULK1-S757, relative to MEFs refed with serum-free DMEM at physiological pH 7.4. Importantly, Torin1 ablated the pHi-induced phosphorylation of these targets (Fig. 1, *B* and *C*). These results demonstrate that an alkaline pHe is sufficient to increase mTORC1 signaling in the absence of serum growth factors. In these experiments, we noted that serum-free media (SFM) at alkaline pH also increased the phosphorylation of Akt T308 (a direct target of PI3K-dependent PDK1) and Akt S473 (a direct target of mTORC2) (Fig. 1, *A*–*C*). Consistent with mTORC2-mediated Akt S473 phosphorylation stabilizing Akt T308 phosphorylation (23, 36, 47), media at alkaline pH increased Akt P-T308 in a Torin1-sensitive manner (Fig. 1, *A*–*C*). We also found that refeeding serum-starved MEFs with media adjusted to increasingly basic or acidic pH values increased (Fig. 1*D*) or decreased (Fig. 1*E*) mTORC1 and mTORC2 signaling, respectively. Finally, we analyzed how SFM at alkaline pH controls mTORC1 and mTORC2 signaling in other serum-starved cell lines, including C2C12, U2OS, HeLa, and human embryonic kidney 293 (HEK293) cells (Fig. S1, *A*–*D*). In C2C12 and U2OS cells, SFM at an alkaline pH increased both mTORC1 (S6K1 P-T389) and mTORC2 (Akt P-S473) signaling (Fig. S1, *A* and *B*). In serum-starved HEK293 cells, SFM at an alkaline pH increased mTORC1 signaling, albeit modestly, and mTORC2 signaling (Fig. S1*C*), whereas in serum-starved HeLa cells, SFM at an alkaline pH increased mTORC1 but not mTORC2 signaling (Fig. S1*D*). Taken together, these results indicate that media at an alkaline pH increases mTORC1 and/or mTORC2 signaling in several cell lines, including primary MEFs, during growth factor limitation, albeit to different degrees for reasons unclear at this time.

### Alkaline pHi upregulates mTORC1 and mTORC2 signaling in the absence of serum growth factors

**Figure 2:**
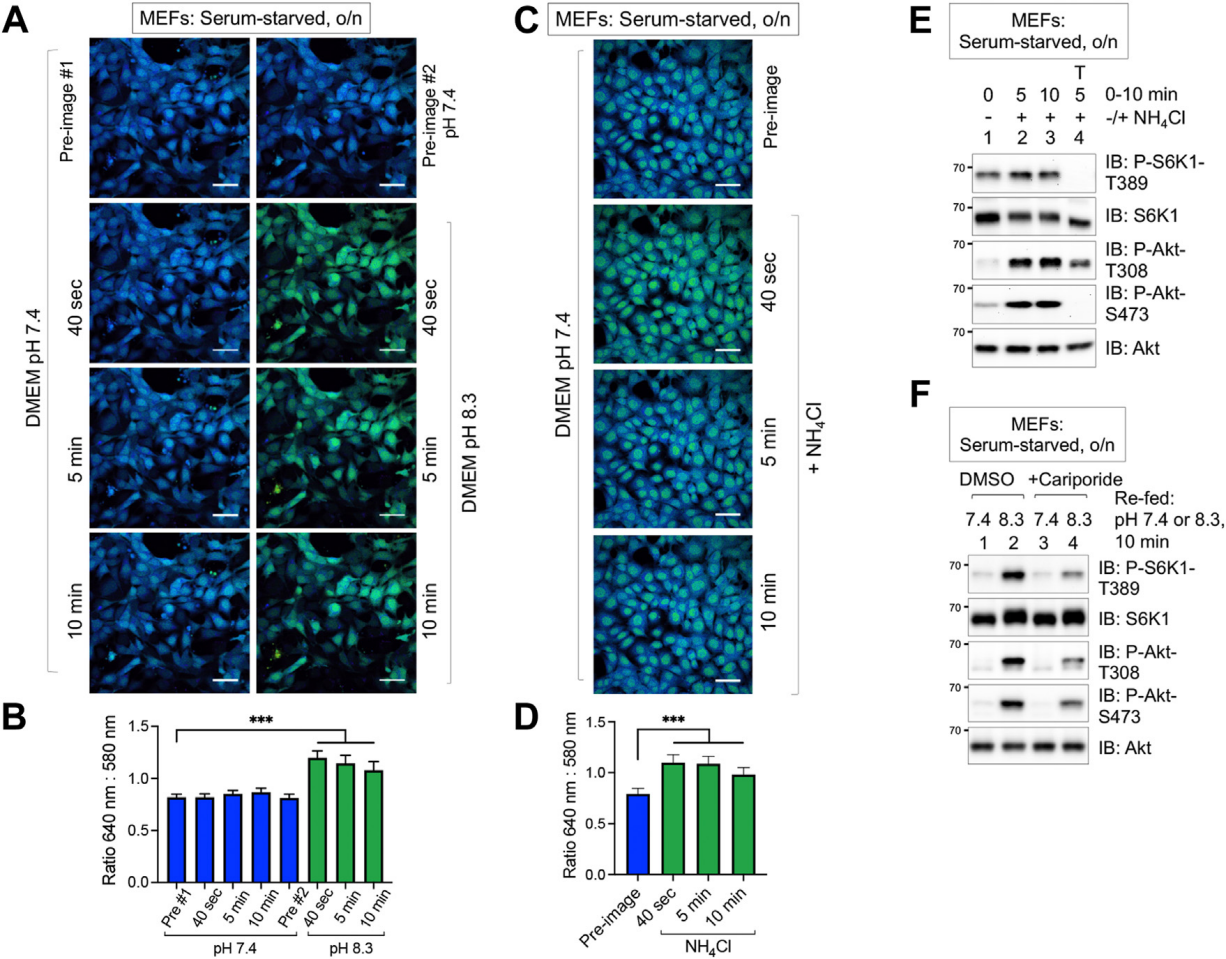
**Incubation of cells in media at alkalineextracellular pHor containing NH**_**4**_**Cl increasesintracellular pH.***A*, MEFs were serum-starved overnight (o/n), incubated in serum-free DMEM (pH 7.4) supplemented with cSNARF1-AM (10 mM) (30 min), and refed with serum-free DMEM (pH 7.4). One image was acquired immediately (preimage #1), and three additional images were acquired at 40 s, 5 min, and 10 min. Cells were next refed with serum-free DMEM (pH 7.4), and one image was acquired immediately (preimage #2). Cells were refed again with serum-free DMEM (pH 8.3), and three images were acquired at 40 s, 5 min, and 10 min. Scale bar represents 50 μm. *B*, quantitation of the results in *A*. Data represent mean ± SD of n = 100 cells from one representative experiment; one-way ANOVA, ∗∗∗*p* < 0.001. *C*, MEFs were loaded with cSNARF1-AM as in *C*. After refeeding with serum-free DMEM pH 7.4, one image was acquired immediately (preimage), at which point NH_4_Cl (10 mM) was added, and three additional images were acquired at 40 s, 5 min, and 10 min. Scale bar represents 50 μm. *D*, quantitation of the results in *C*. Data represent mean ± SD (n = 20 cells from one representative experiment); one-way ANOVA, ∗∗∗*p* < 0.001. All panels show representative results from at least three independent experiments. *E*, MEFs were serum-starved o/n, pretreated without or with Torin1 (T) (100 nM) (30 min), and treated without or with NH_4_Cl (10 mM) (5 and 10 min). *F*, MEFs were serum-starved o/n, pretreated without (DMSO) or with cariporide (10 μM) (30 min), and refed with serum-free DMEM at pH 7.4 or set to pH 8.3 in the absence or the presence of cariporide (10 min). All panels show representative results from at least three independent experiments. Molecular weight markers are provided in kilodalton. DMEM, Dulbecco’s modified Eagle’s medium; DMSO, dimethyl sulfoxide; MEF, mouse embryonic fibroblast.

Prior work by other groups and us demonstrated that refeeding cells with serum-containing CM at an alkaline pH raises pHi rapidly (41, 48, 49). To confirm that culturing serum-starved MEFs in SFM at an alkaline pH also raises pHi rapidly, we used live-cell imaging coupled with a cell-permeable and pH-sensitive fluorescent dye (cSNARF1-AM), which monitors changes in pHi between 7 and 8. cSNARF1-AM undergoes a pH-sensitive shift in fluorescence wavelength emission depending on its protonation state. With 488 nm excitation, peak emission occurs at 580 nm in the protonated state (more acidic conditions) and 640 nm in the deprotonated state (more alkaline conditions). Thus, ratiometric imaging (640:580 nm) enables the detection of changes in pHi within the physiological range of normal cells (∼pH 7.05–7.2) and cancer cells (∼pH 7.3–7.6) (42, 43, 44, 45). Serum-starved MEFs were loaded with cSNARF1-AM, washed, refed with SFM at pH 7.4 or pH 8.3, and imaged at 0, 0.67 (40 s), 5, and 10 min. Visualization of the acquired pseudocolored ratiometric images (640:580 nm) and quantitation of the signal ratios revealed a rapid and significant increase in pHi upon shift to serum-free DMEM at alkaline pH (*green cells*) relative to those maintained in media at physiological pH (*blue cells*) (Fig. 2, *A* and *B*). These results demonstrate that shifting cells to SFM at alkaline pH leads to a rapid rise in pHi.

As an alternative method to raising pHi, we used ammonium chloride (NH_4_Cl). NH_4_Cl increases cytosolic pH upon rapid diffusion of NH_3_ (a weak base) into the cell and conversion to ammonium ion (NH_4_^+^) by scavenging protons from the cytosol, thus increasing cytosolic pH (50, 51). Slower and subsequent entry of NH_4_^+^ into the cell attenuates the rise in pH upon its dissociation into NH_3_ and H^+^. As NH_4_Cl raises the lumenal pH of organelles such as lysosomes, it is often employed in autophagy research to impair autophagic flux/proteolysis, which requires an acidic pH in autolysosomes (50, 51). The addition of NH_4_Cl to SFM at pH 7.4 induced a rapid but transient rise in pHi (Fig. 2, *C* and *D*), as measured microscopically with cSNARF1-AM. Biochemically, treatment of serum-starved MEFs with NH_4_Cl increased mTORC1 signaling weakly (S6K1 P-T389) but increased PI3K (Akt P-T308) and mTORC2 (Akt P-S473) signaling strongly (Fig. 2*E*).

We next sought to confirm that culturing cells in SFM at an alkaline pH increases mTORC1 and mTORC2 signaling by raising pHi. To do so, we used the drug cariporide (a more-specific derivative of amiloride) to reduce pHi pharmacologically. Cariporide inhibits NHE1 (sodium-hydrogen exchanger isoform 1), a sodium–proton (Na^+^/H^+^) antiporter on the plasma membrane that pumps hydrogen ions (H^+^) from the cytosol to the extracellular space. Thus, cariporide decreases pHi by blunting H^+^ efflux (52, 53, 54). We, therefore, pretreated serum-starved MEFs with cariporide before refeeding cells with media at alkaline pH in the absence or the presence of drug. Cariporide blunted the ability of media at pH 8.3 to increase the phosphorylation of S6K1 T389, Akt T308, and Akt S473 (Fig. 2*F*). These results indicate that attenuating H^+^ efflux from the cell suppresses mTORC1 and mTORC2 signaling likely by acidifying the cytosol, thus countering the alkalizing effect of media at pH 8.3. Note that NHEI represents one of many hydrogen ion transporters in cells, thus explaining why cariporide only partly blunts the activation of mTORC1 signaling in response to alkaline pHi. Taken together, these results indicate that altering pHi by three independent methods (treating cells with media at alkaline pH, NH_4_Cl, or cariporide) modulates mTORC1 and mTORC2 signaling in the absence of serum growth factors, with alkaline pHi or acidic pHi increasing or decreasing mTORC1/2 signaling, respectively.

### Alkaline pHi mimics growth factor signaling to activate mTORC1 in parallel to amino acid signaling

**Figure 3:**
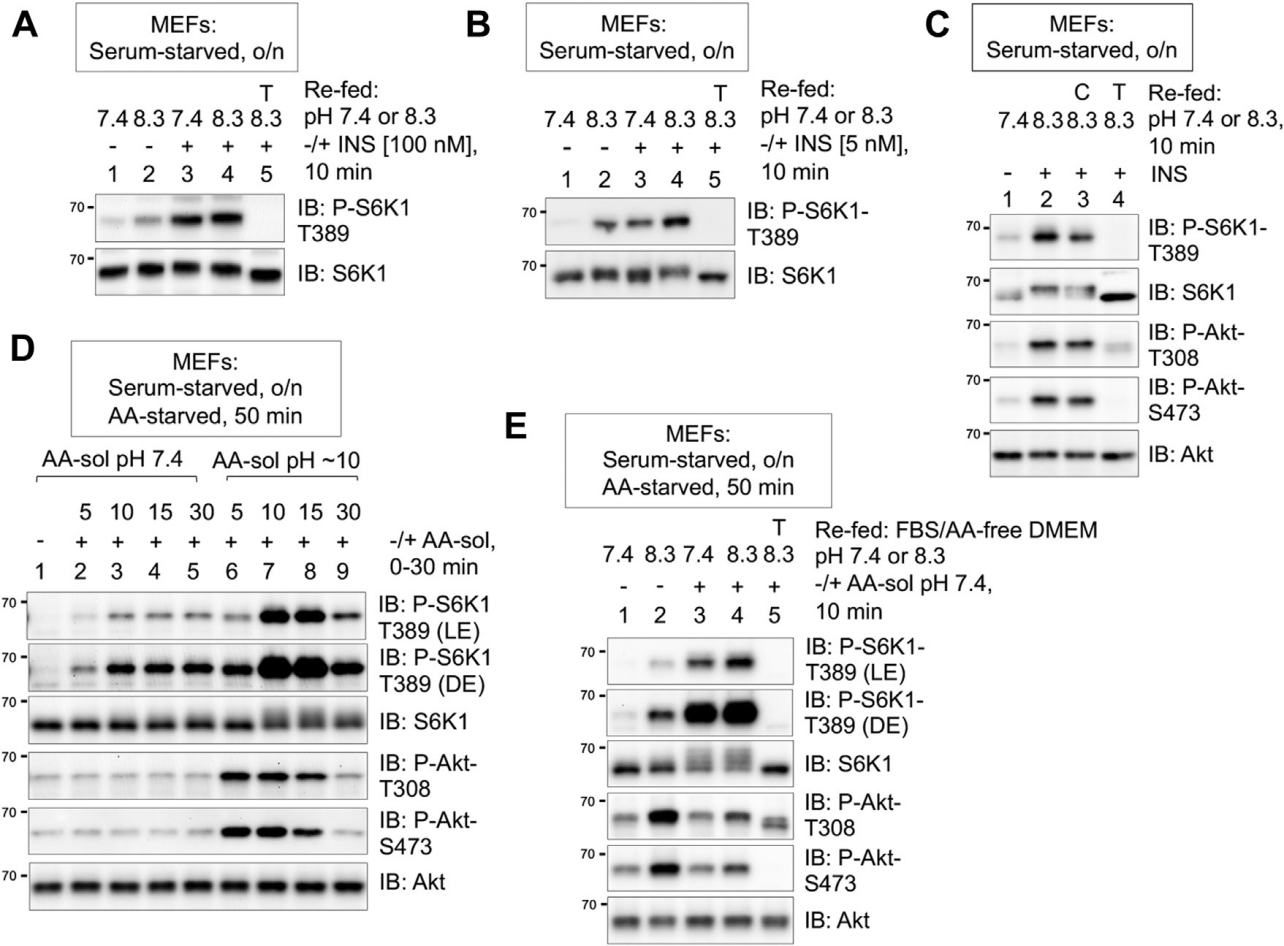
**Alkaline intracellular pH (pHi) mimics growth factor signaling to activate mTORC1 in parallel to amino acid signaling.***A*, MEFs were serum-starved overnight (o/n) and pretreated without or with Torin1 (T) (100 nM) (30 min), refed with serum-free DMEM at pH 7.4 or pH 8.3 without (−) or with (+) 100 nM insulin (10 min) in the absence or the presence of Torin1 (T). Whole-cell lysates were immunoblotted as indicated. *B*, MEFs were treated as in *A*, except that cells were stimulated with a submaximal dose of insulin, 5 nM. *C*, MEFs were serum-starved o/n, amino acid-starved for 50 min in DMEM lacking amino acids and FBS, and then stimulated without (−) or with (+) an amino acid solution (1× final) whose pH was adjusted to 7.4 or not adjusted (Ph = ∼10) (0–30 min). Note: The concentration of amino acids added to (1×) is similar to those found in RPMI media. *D*, MEFs were deprived of serum growth factors and amino acids as in *C*, pretreated without or with Torin1 (100 nM) (30 min), and refed with serum- and amino acid–free DMEM at pH 7.4 or pH 8.3 without (−) or with (+) AAs (1×) (10 min). *E*, MEFs were serum-starved o/n and pretreated without or with cariporide (C) (10 μM) and Torin1 (T) (100 nM) (30 min), refed with serum-free DMEM at pH 7.4 or pH 8.3 without (−) or with (+) 100 nM insulin (10 min) in the absence or the presence of drugs. Whole-cell lysates were immunoblotted as indicated. All panels show representative results from at least three independent experiments. Molecular weight markers are provided in kilodalton. AA-sol, amino acid solution; DE, dark exposure; DMEM, Dulbecco’s modified Eagle’s medium; FBS, fetal bovine serum; LE, light exposure; MEF, mouse embryonic fibroblast; mTORC1, mechanistic target of rapamycin complex 1.

Growth factors and amino acids activate mTORC1 cooperatively through parallel pathways, although some crosstalk occurs. We, thus, next investigated the relationship between mTORC1 signaling induced by alkaline pHi *versus* insulin or amino acids. Treatment of serum-starved MEFs with SFM at pH 8.3 or with a maximal dose of insulin (100 nM) each increased S6K1 P-T389, whereas the addition of both together failed to increase S6K1 P-T389 further (Fig. 3*A*). When we stimulated cells with a submaximal dose of insulin (5 nM), however, alkaline pHi and insulin increased S6K1 P-T389 in an additive manner (Fig. 3*B*). Taken together, these data are consistent with the idea that alkaline pHi mimics growth factor signaling. As insulin and growth factors were shown several decades ago to increase pHi in fibroblasts and frog skeletal muscle by activating NHEI (55, 56, 57, 58, 59, 60, 61), we next asked whether blunting H^+^ extrusion from the cell through inhibition of NHEI with cariporide would blunt the activating effect of insulin on mTORC1 and mTORC2 signaling. We found that cariporide pretreatment blunted the ability of insulin to increase mTORC1 signaling, albeit modestly. Cariporide had minimal effect on mTORC2 signaling, however (Fig. 3*C*).

We next investigated the relationship between mTORC1 signaling induced by alkaline pHi *versus* amino acids. We first tested how the addition of the commercial amino acid solution adjusted to pH 7.4 increases mTORC1 signaling relative to the addition of the original, unadjusted pH ∼10 commercial amino acid solution. As expected, after starving MEFs of both growth factors and amino acids, amino acids at pH 7.4 increased S6K1 P-T389 but not Akt P-S473; amino acids at pH ∼10 increased S6K1 P-S6K1 much more strongly, however, and also increased Akt P-S473 (Fig. 3*D*). These results suggest that alkaline pHi and amino acid signaling increase mTORC1 signaling additively by parallel pathways. Moreover, they demonstrate that alkaline pHi but not amino acids increase mTORC2 signaling. To analyze the effects of amino acids *versus* an alkaline pHi on mTORC1 signaling more carefully, we tested their ability alone and together to increase mTORC1 signaling. Refeeding serum- and amino acid-starved MEFs with media at pH 8.3 in the presence of amino acids increased mTORC1 signaling to a greater extent than either stimulus alone (Fig. 3*E*) (lane 4 *versus* lanes 2 and 3). These results suggest that amino acids and alkaline pHi signal in parallel to promote mTORC1 signaling. Interestingly, amino acid stimulation attenuated the ability of an alkaline pHi to increase in Akt P-T308 and P-S473 (lane 4) (Fig. 3*E*). Perhaps the well-established ability of mTORC1 to mediate negative feedback on PI3K flux (62, 63, 64) accounts for this effect, although 10 min would be quite fast for such a mechanism. Taken together, these results indicate that alkaline pHi mimics growth factor–PI3K signaling and therefore cooperates with amino acid signaling to fully activate mTORC1. In addition, they underscore the importance of controlling the pHe in cell cultures, as the results in Figure 3*D* could be interpreted to mean, incorrectly, that amino acids increase mTORC2 signaling. Failure to control the pH of commercial amino acid solutions likely explains why some studies concluded that amino acids activate mTORC2 in certain contexts (65, 66).

### mTORC1 and mTORC2 signaling induced by alkaline pHi requires PI3K activity

**Figure 4:**
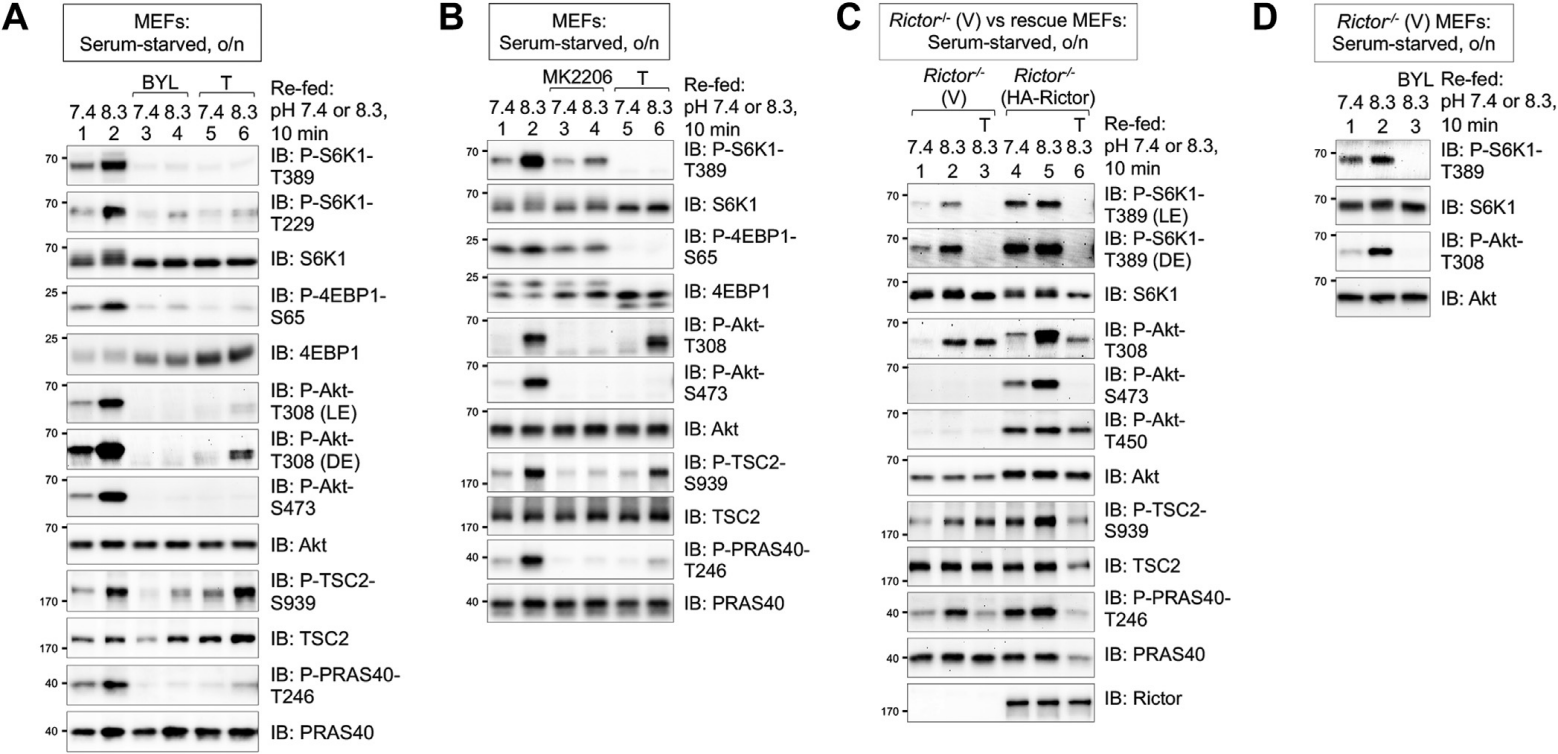
**mTORC1 and mTORC2 signaling induced by alkaline pHi requires PI3K activity.***A*, MEFs were serum-starved overnight (o/n), pretreated without or with BYL719 (BYL) (10 μM) or Torin1 (T) (100 nM) (30 min), and refed with serum-free DMEM at pH 7.4 or pH 8.3 in the absence or the presence of drugs. Whole-cell lysates were immunoblotted as indicated. *B*, MEFs were treated as in *A*, except they were pretreated with MK2206 (10 μM) instead of BYL719 (30 min). *C*, *Rictor*^*−/−*^ MEFs expressing vector control (V) or rescued with HA-Rictor were serum-starved o/n, pretreated without or with Torin1 (T) (100 nM) (30 min), and refed with serum-free DMEM at pH 7.4 or pH 8.3 (10 min). *D*, *Rictor*^*−/−*^ MEFs expressing vector control (V) were treated as in *C*, except they were pretreated with BYL719 (10 μM) (30 min). All panels show representative results from at least three independent experiments. Molecular weight markers are provided in kilodalton. DE, dark exposure; DMEM, Dulbecco’s modified Eagle’s medium; HA, hemagglutinin; LE, light exposure; MEF, mouse embryonic fibroblast; mTORC1, mechanistic target of rapamycin complex 1; mTORC2, mechanistic target of rapamycin complex 2; pHi, intracellular pH; PI3K, phosphatidylinositide 3-kinase.

To ask whether the ability of alkaline pHi to increase mTORC1 signaling requires PI3K, we treated serum-starved MEFs with BYL719 prior to incubation in SFM at pH 8.3. BYL719 blocked the ability of alkaline pHi to increase the phosphorylation of S6K1-T389, 4EBP1-S65, and Akt-S473 (Fig. 4*A*). BYL719 also blocked the PDK1-dependent phosphorylation of the activation loop sites on S6K1 (P-T229) and Akt (P-T308) as well as the phosphorylation of the Akt substrates TSC2-S939 and proline-rich Akt substrate of 40 kDa (PRAS40)-T246 (Fig. 4*A*). The Akt inhibitor MK2206 blocked the ability of alkaline pHi to increase the phosphorylation of S6K1-T389, 4EBP1-S65, Akt-T308, TSC2-S939, and PRAS40-T246 (Fig. 4*B*), thus phenocopying the effect of PI3K inhibition. Collectively, these results indicate that PI3K–Akt signaling is required for the activation of mTORC1 and mTORC2 signaling by alkaline pHi in the absence of serum growth factors.

Phosphorylation of Akt S473 by mTORC2 often increases Akt T308 phosphorylation, possibly by recruiting PDK1 and/or stabilizing Akt T308 phosphorylation (23, 47), whereas cotranslational phosphorylation of Akt T450 by mTORC2 increases Akt stability (67, 68). As expected, Torin1 not only ablated Akt P-S473 in cells cultured in media at both pH 7.4 and pH 8.3 but also reduced alkaline pHi–induced Akt P-T308 as well as PRAS40 P-T246, an Akt substrate (Figs. 4, *A*–*C* and 1, *A* and *B*). It was therefore important to determine whether the ability of alkaline pHi to increase mTORC1 signaling occurs independently of the activating effect of alkaline pHi on mTORC2 signaling or not. We thus studied *Rictor*^*−/−*^ MEFs stably expressing either vector control or rescued with hemagglutinin (HA)-tagged Rictor. Restoring mTORC2 function boosted S6K1 P-T389 overall in rescued *Rictor*^*−/−*^ MEFs refed with SFM at either pH 7.4 or 8.3 (Fig. 4*C*) (likely because of the combined effect of elevated Akt P-T308 and total Akt protein levels). In *Rictor*^*−/−*^ MEFs expressing vector control (V) and lacking mTORC2-dependent Akt P-S473, alkaline pHi still increased PI3K signaling to Akt (P-T308), TSC2 (P-S939), and PRAS40 (P-T246) as well as mTORC1 signaling to S6K1 (P-T389) (Fig. 4*C*). In this context, Torin1 had no inhibitory effect on Akt P-T308 or TSC2 P-S939 (Fig. 4*C*). In *Rictor*^*−/−*^ (V) MEFs, BYL719 blocked the ability of media at pH 8.3 to increase S6K1 P-T389 and Akt P-T308 (Fig. 4*D*). Collectively, these results demonstrate that alkaline pHi increases the PI3K-dependent activation of Akt and mTORC1 in the absence of mTORC2 function.

As driver mutations in *PIK3Cα*, which encodes the catalytic p110 subunit of class I PI3Kα, promote the growth of many cancers including breast cancer because of elevated PI3K activity, we tested whether breast cancer MCF7 cells bearing an oncogenic mutation in *PIK3CA* (E545K) would be resistant to the ability of an alkaline pHi to increase mTORC1 and/or mTORC2 signaling. As expected, serum-starved MCF7 cells displayed an easily detectable level of BYL719-sensitive mTORC1 (S6K1 P-T389) and mTORC2 (Akt P-S473) signaling because of elevated PI3K activity. In this context, an alkaline pHi failed to increase mTORC1 or mTORC2 signaling (Fig. S2). These results support the idea that an alkaline pHi mimics PI3K signaling further.

### Alkaline pHi dissociates TSC2 from lysosomal membranes and increases mTORC1 catalytic activity by increasing PI3K activity

**Figure 5:**
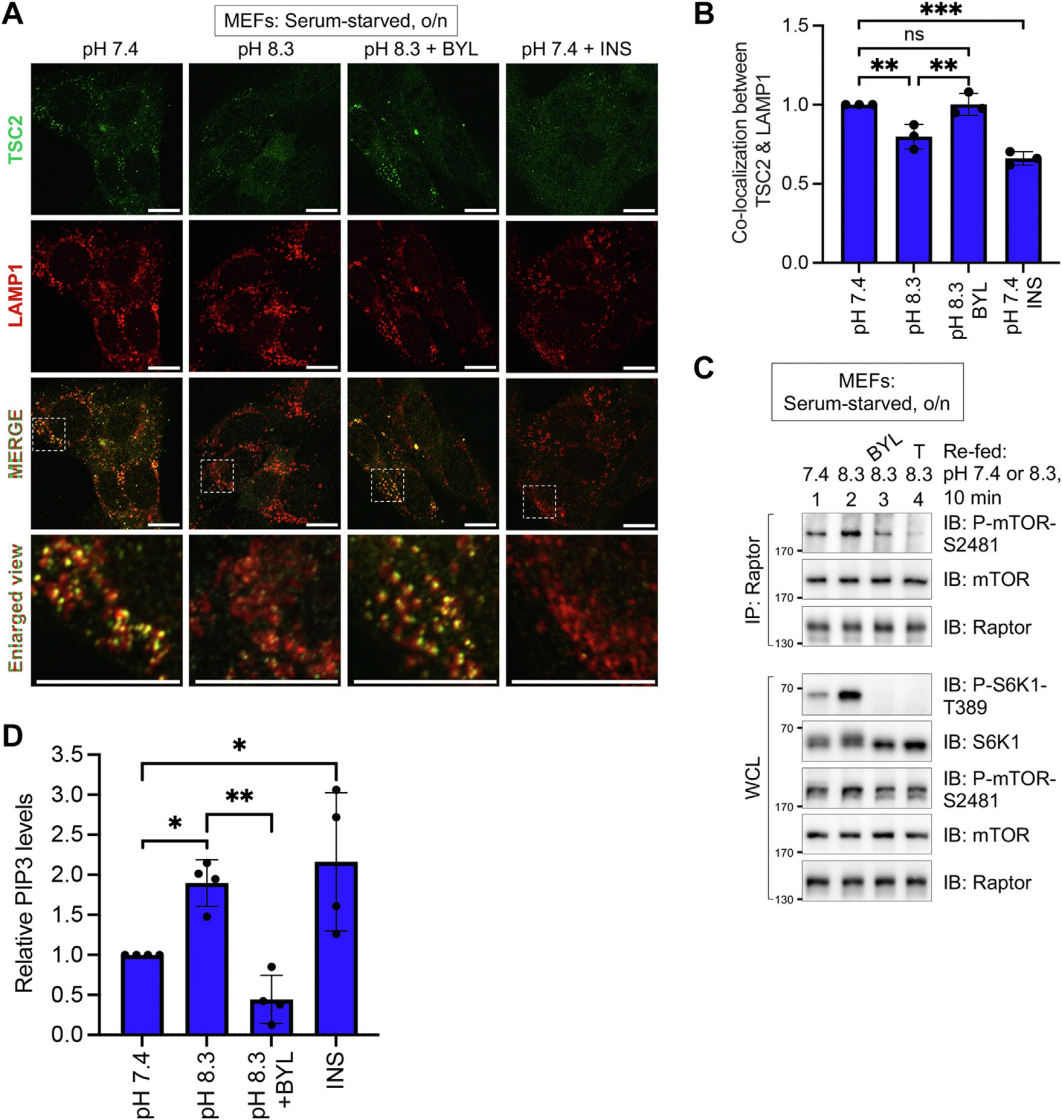
**Alkaline pHi dissociates TSC2 from lysosomal membranes and increases mTORC1 catalytic activity by increasing PI3K activity**. *A*, MEFs were serum-starved overnight (o/n), pretreated without or with BYL719 (BYL) (10 μM) (30 min), and refed with serum-free DMEM (pH 7.4 or pH 8.3). Cells were also stimulated with insulin (100 nM) (10 min), as indicated. Cells were then fixed and processed for confocal immunofluorescence microscopy using anti-TSC2 (*green*) and anti-LAMP1 (*red*) antibodies, and the nuclei were stained with DAPI (not shown). Single channels for TSC2 and LAMP1 are shown as well as a merged channel. Scale bar represents 10 μm. *B*, colocalization between TSC2 and LAMP1 was quantified using Pearson’s correlation coefficient. The graph represents mean ± SD of n = 3 independent experiments whereby colocalization was measured in 10 fields of cells (∼20 cells/field) in experiment 1, 10 fields of cells (∼20 cells/field) in experiment 2, and five fields of cells (∼20 cells/field) in experiment 3; one-way ANOVA. ∗∗*p* < 0.01, ∗∗∗*p* < 0.001. *C*, MEFs were treated as in *A*. Raptor was immunoprecipitated, and immunoprecipitates (IPs) and whole-cell lysates (WCLs) were immunoblotted as indicated. *D*, MEFs were treated as in *A*. PIP_3_ on total membranes was extracted and analyzed by ELISA. Data represent mean ± SD; n = 4 independent experiments; one-way ANOVA, ∗*p* < 0.05, ∗∗*p* < 0.01. All panels show representative results from at least three independent experiments. Molecular weight markers are provided in kilodalton. DAPI, 4′,6-diamidino-2-phenylindole; LAMP1, lysosomal-associated membrane protein 1; MEF, mouse embryonic fibroblast; mTORC1, mechanistic target of rapamycin complex 1; ns, not significant; pHi, intracellular pH; PI3K, phosphatidylinositide 3-kinase; PIP_3_, PI-3,4,5-P3; TSC2, tuberous sclerosis complex 2.

We next sought to better define the mechanism by which alkaline pHi increases mTORC1 signaling. As activation of Akt by PI3K dissociates the TSC complex from lysosomal membranes through Akt-mediated inhibitory phosphorylation of TSC2 (15), we asked whether alkaline pHi is sufficient to dissociate TSC2 from lysosomal membranes in the absence of growth factors. Refeeding serum-starved MEFs with media at pH 8.3 decreased colocalization of TSC2 with lysosomal-associated membrane protein 1 (LAMP1), a lysosomal membrane marker, in a BYL719-sensitive manner (Fig. 5, *A* and *B*). As expected, insulin stimulation of serum-starved MEFs at physiological pH reduced the colocalization of TSC2 with LAMP1, as reported previously (Fig. 5, *A* and *B*) (15). Thus, alkaline pHi is sufficient to dissociate TSC2 from lysosomal membranes in the absence of serum growth factors. We next investigated whether alkaline pHi increases the intrinsic catalytic activity of mTORC1. To do so, we monitored levels of Raptor-associated mTOR S2481 autophosphorylation, a simple method to monitor the catalytic activity of mTORC1 in intact cells (69). Refeeding MEFs with media at pH 8.3 increased P-S2481 on Raptor-associated mTOR, which occurred in a BYL719- and Torin1-sensitive manner (Fig. 5*B*). As the ability of alkaline pHi to dissociate TSC2 from lysosomes and increase mTORC1 activity and signaling was PI3K dependent, we next asked whether alkaline pHi increases PI3K activity. We refed serum-starved MEFs with SFM at pH 7.4 or pH 8.3 and measured the levels of PIP_3_ in purified lipids by an ELISA. As a positive control, we stimulated cells in media at pH 7.4 with insulin. Media at pH 8.3 increased PIP_3_ levels relative to media at pH 7.4 in a statistically significant and BYL719-sensitive manner (Fig. 5, *C* and *D*). As expected, insulin increased PIP_3_ levels. These results indicate that alkaline pHi is sufficient to increase PI3K activity in the absence of growth factors. Taken together, they reveal that by increasing PI3K activity and Akt signaling, alkaline pHi dissociates TSC2 from the lysosomal surface, thus enabling Rheb to increase mTORC1 catalytic activity.

### Alkaline pHi increases cap-dependent protein synthesis and suppresses apoptosis in a PI3K- and mTOR-dependent manner in the absence of serum growth factors

**Figure 6:**
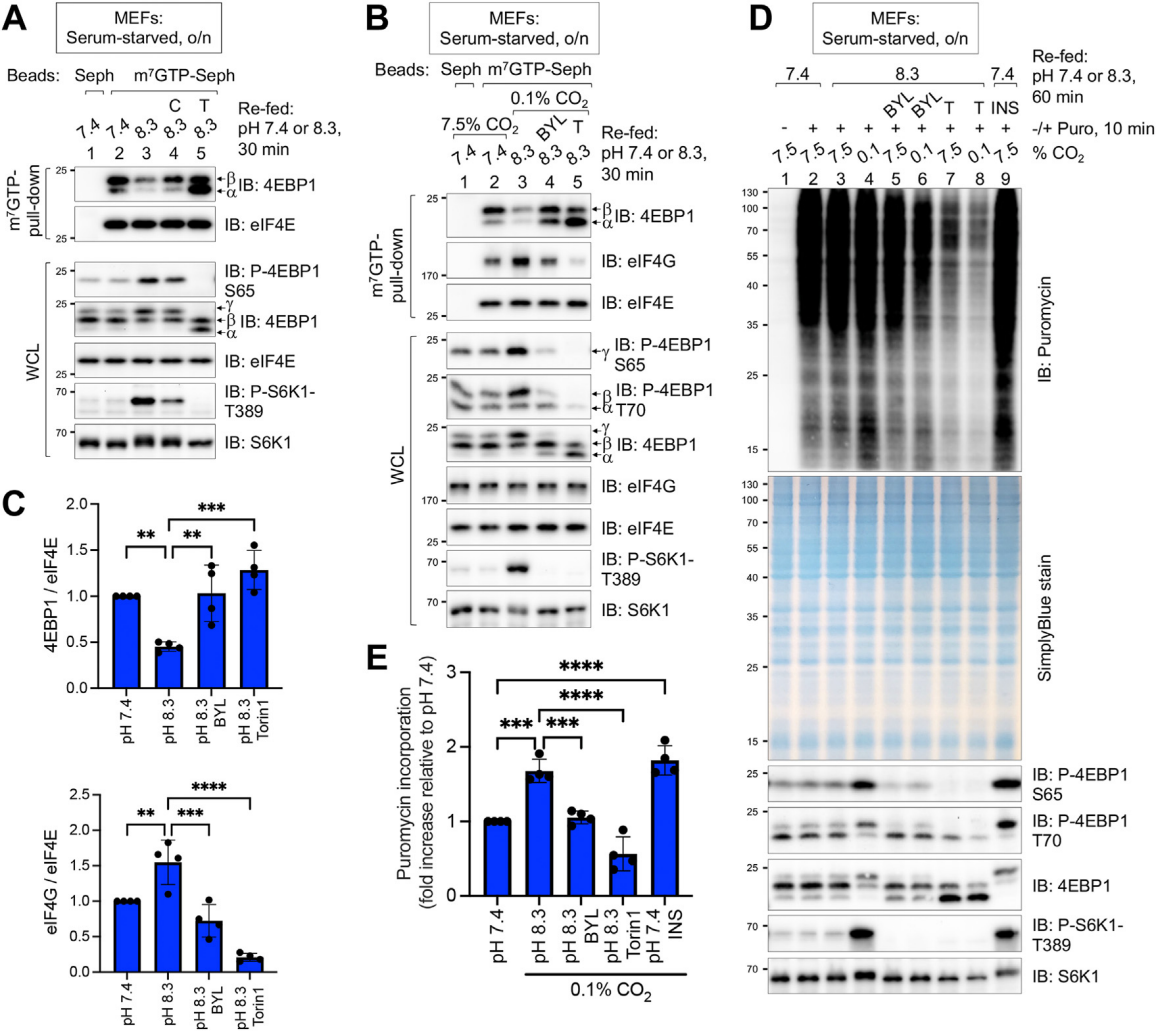
**Alkaline pHi increases cap-dependent protein synthesis in the absence of growth factors through PI3K–mTOR signaling.***A*, MEFs were serum-starved overnight (o/n), pretreated without or with cariporide (C) (10 μM) or Torin1 (T) (100 nM) (30 min), and refed with serum-free DMEM at pH 7.4 or 8.3 in the absence or th presence of drugs (30 min). eIF4E was pulled down with 7-methyl GTP-Sepharose beads. Pull-downs and whole-cell lysates were immunoblotted as indicated. *B*, MEFs were treated as in *A*, except they were pretreated with BYL719 (BYL) (10 μM) or Torin1 (100 nM) (30 min) and incubated at either normal (7.5%) or low CO_2_ (0.1%). eIF4E was pulled down with 7-methyl GTP-Sepharose beads. Pull-downs and whole-cell lysates were immunoblotted as indicated. Note: Low CO_2_ maintains the media at an alkaline pH for a longer period. *C*, quantitation of the results shown in *B*, that is, the amount of 4EBP1 (*left graph*) or eIF4G (*right graph*) that coprecipitates with eIF4E at pH 7.4 *versus* pH 8.3. Data represent mean ± SD (n = 4 independent experiments; one-way ANOVA; ∗∗*p* < 0.01; ∗∗∗*p* < 0.001; ∗∗∗∗*p* < 0.0001. *D*, MEFs were treated as in *A*, except they were refed with DMEM at pH 7.4 or pH 8.3 for 60 min (instead of 10 min) and pulsed with puromycin (10 μg/ml) for the last 10 min. Cells were cultured in incubators set to either 7.5% CO_2_ or 0.1% CO_2_. Note: Low CO_2_ maintains the media at an alkaline pH for a longer period. *E*, quantitation of puromycin incorporation. Data represent mean ± SD (n = 4 independent experiments); one-way ANOVA; ∗∗∗*p* < 0.001; ∗∗∗∗*p* < 0.0001. All panels show representative results from at least three independent experiments. Molecular weight markers are provided in kilodalton. 4EBP1, eIF4E binding protein 1; DMEM, Dulbecco’s modified Eagle’s medium; eIF4E, eukaryotic initiation factor 4E; MEF, mouse embryonic fibroblast; mTOR, mechanistic target of rapamycin; pHi, intracellular pH; PI3K, phosphatidylinositide 3-kinase.

As protein synthesis represents a major cellular function promoted by mTORC1 (33, 34), we next asked whether alkaline pHi is sufficient to increase cap-dependent protein synthesis in the absence of serum growth factors through PI3K and mTOR. We first pulled down eIF4E using m^7^GTP-coupled Sepharose beads to assess the ability of alkaline pHi to dissociate 4EBP1 from eIF4E through mTORC1-mediated 4EBP1 phosphorylation. After serum starvation, refeeding MEFs with serum-free DMEM at pH 8.3 was sufficient to increase 4EBP1 phosphorylation and dissociate 4EBP1 from eIF4E, which occurred in a Torin1-sensitive manner (Fig. 6*A*). Pretreating cells with cariporide to suppress H^+^ efflux and thus reduce pHi blunted the ability of alkaline pHi to dissociate 4EBP1 from eIF4E. As the pH of DMEM at pH 8.3 falls quickly because of the strong buffering capacity of DMEM incubated in an atmosphere of 7.5% CO_2_, we incubated cells at low CO_2_ (*i.e.*, 0.1%) to maintain an elevated pH for a longer period. This modification to our method for raising pHi enabled us to study protein synthesis (see later), a process requiring a longer amount of time. In cells cultured at low CO_2_, alkaline pHi dissociated 4EBP1 from eIF4E and increased the association of eIF4G with eIF4E in a statistically significant and PI3K- and mTOR- dependent manner (Fig. 6, *B* and *C*). As in other experiments, alkaline pHi increased the PI3K- and mTOR-dependent phosphorylation of 4EBP1 and S6K1 in cells cultured at low CO_2_ in this context (Fig. 6*B*). These results indicate that elevated pHi inhibits 4EBP1 function and promotes the assembly of translation preinitiation complexes.

We next asked whether increased assembly of eIF4E-based translation preinitiation complexes leads to increased protein synthesis. To do so, we employed the nonradioactive SUnSET assay, which measures the incorporation of the tRNA-mimetic compound puromycin into nascent polypeptide chains by simple Western blotting using an antipuromycin antibody (70). Upon refeeding serum-starved MEFs incubated at either normal CO_2_ (*i.e.*, 7.5%) or low CO_2_ (*i.e.*, 0.1%) with serum-free DMEM at pH 7.4 or pH 8.3 for 60 min that lacked or contained a pulse of puromycin for the last 10 min, we found that alkaline pHi was sufficient to increase protein synthesis in a statistically significant manner at low CO_2_, which was PI3K and mTOR dependent (Fig. 6, *D* and *E*). As expected, stimulation of serum-starved cells cultured at physiological pH 7.4 with insulin also increased protein synthesis (Fig. 6, *D* and *E*). Consistently, alkaline pHi increased the PI3K- and mTOR-dependent phosphorylation of 4EBP1 and S6K1 T389 in this experimental context.

**Figure 7:**
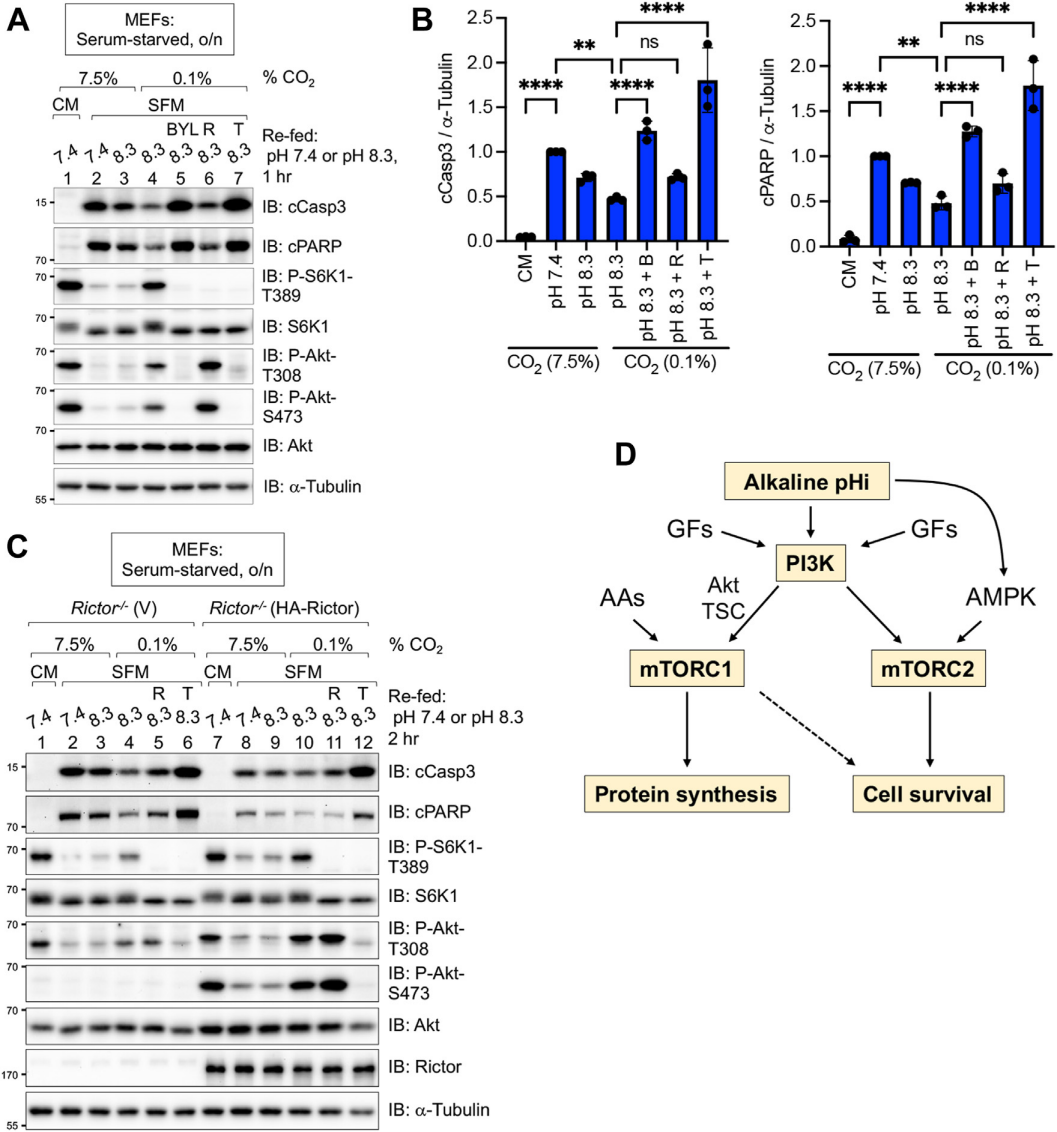
**Alkaline intracellular pH (pHi) suppresses apoptosis in the absence of growth factors through PI3K–mTOR signaling.***A*, MEFs were cultured either in complete media (CM) (DMEM/FBS) (lane 1 only) or serum-free media (SFM) (DMEM lacking FBS) overnight (o/n) (16 h) (lanes 2–7), pretreated without or with BYL719 (BYL) (10 mM), rapamycin (R) (20 ng/ml = 22 nM), or Torin1 (T) (100 nM) (30 min), and then refed for 1 h with either CM (lane 1) or SFM at pH 7.4 or pH 8.3 (lanes 2–7). Cells were cultured in incubators set to either 7.5% or 0.1% CO_2_, as indicated. Whole-cell lysates were immunoblotted as indicated. Note: Low CO_2_ maintains the media at an alkaline pH for a longer period. *B*, levels of cleaved caspase 3 (cCasp3) (*left graph*) and cleaved poly(ADP-ribose) polymerase (cPARP) (*right graph*) relative to total α-tubulin in *A* were quantitated. n = 3 independent experiments; one-way ANOVA; ∗∗*p* < 0.01; ∗∗∗*p* < 0.001; and ∗∗∗∗*p* < 0.0001. *C*, *Rictor*^*−/−*^ MEFs rescued with vector control (V) or HA-Rictor were treated as in *A*, except they were pretreated with rapamycin (20 ng/ml) (30 min) or Torin and incubated for 2 h (instead of 1 h) in SFM at pH 7.4 or 8.3. *D*, model. Alkaline pHi increases PI3K activity, which upregulates mTORC1 and mTORC2 signaling to increase cap-dependent protein synthesis and cell survival during growth factor limitation. All panels show representative results from at least three independent experiments. Molecular weight markers are provided in kilodalton. DMEM, Dulbecco's modified Eagle's medium; FBS, fetal bovine serum; HA, hemagglutinin; MEF, mouse embryonic fibroblast; mTORC1, mechanistic target of rapamycin complex 1; mTORC2, mechanistic target of rapamycin complex 2; PI3K, phosphatidylinositide 3-kinase.

Cell survival represents an important cellular function mediated by mTORC2 and Akt (5, 12). Our previous work demonstrated that an AMPK–mTORC2 signaling axis suppresses apoptosis in response to energetic stress (36) or alkaline pHi (41). We, therefore, tested whether activation of PI3K–mTOR signaling by alkaline pHi promotes cell survival during growth factor deprivation. We incubated MEFs in either CM (*i.e.*, DMEM containing FBS) (lane 1) or in SFM (*i.e.*, DMEM lacking FBS) (lane 2) overnight (16 h) and then refed the cells for 1 h with either CM (lane 1) or SFM at pH 7.4 or pH 8.3 (lanes 2–7). As expected, cells cultured in SFM at pH 7.4 for the full 17 h displayed increased apoptosis (lane 2) relative to those kept in CM for 17 h (lane 1), as monitored by Western blotting of cleaved caspase 3 (cCasp3) and cleaved poly(ADP-ribose) polymerase (cPARP), well-established apoptotic markers (Fig. 7, *A* and *B*). Culturing MEFs in SFM at pH 8.3 and 7.5% CO_2_ reduced levels of cCasp3 and cPARP slightly (lane 3). Culturing MEFs in SFM at pH 8.3 and 0.1% CO_2_, however, reduced the levels of cCasp3 and cPARP significantly (lane 4) relative to physiological pH 7.4 (lane 2) and did so in a BYL719- and Torin1-sensitive manner (lanes 5 and 7) (Fig. 7, *A* and *B*). While the mTORC1-specific inhibitor rapamycin blunted the ability of alkaline pHi to suppress apoptosis modestly (lane 6), the effect was not statistically significant (Fig. 7*B*). Consistent with other experiments, alkaline pHi increased PI3K signaling (Akt P-T308), mTORC1 signaling (S6K1 P-T389), and mTORC2 signaling (Akt P-S473) in a PI3K- and mTOR-dependent manner in this experimental context (Fig. 7*A*). Collectively, these results indicate that alkaline pHi protects against apoptosis during growth factor limitation by increasing PI3K–mTOR signaling.

We next asked whether PI3K–mTORC1 signaling induced by alkaline pHi protects against apoptosis during growth factor deprivation in the absence of confounding mTORC2 survival signaling. As before, we analyzed *Ricto*r−/− MEFs stably expressing either vector control (V) or rescued with HA-Rictor. Upon growth factor withdrawal, *Rictor*^*−/−*^ (V) MEFs displayed higher overall levels of cCasp3 and cPARP than rescued *Rictor*^*−/−*^ (HA-Rictor) MEFs (Fig. 7*C*) (lanes 2 *versus* 8). As expected, levels of Akt P-S473 were extremely low in *Rictor*^*−/−*^ (V) MEFs, and reconstitution with HA-Rictor rescued Akt S473 phosphorylation and boosted Akt T308 phosphorylation with little effect on S6K1 T389 phosphorylation (Fig. 7*C*). In *Rictor*^*−/−*^ (V) MEFs cultured in SFM at low CO_2_ (0.1%), alkaline pHi 8.3 increased S6K1 P-T389 and reduced levels of cCasp3 and cPARP relative to cells cultured at physiological pHi 7.4 (lane 4 *versus* lane 2). These effects were both rapamycin and Torin1 sensitive (lanes 5 and 6 *versus* lane 4), with Torin1 having a stronger effect than rapamycin (Fig. 7*C*). These results suggest that in the absence of mTORC2 function, increased mTORC1 signaling in growth factor–starved cells contributes to the evasion of apoptosis mediated by alkaline pHi. Collectively, we propose that activation of both mTORC1 and mTORC2 by alkaline pH promotes cell survival during growth factor withdrawal, with mTORC2 playing a more major role in cell survival than mTORC1.

## Discussion

Normal cells and those associated with various disease states such as cancer display changes in pHi dynamics (42, 43, 44, 45). Normal cells maintain pHi at ∼7.05 to 7.2 through regulation of hydrogen ion (H^+^) dynamics at the plasma membrane and possibly internal membranes (43, 44). Original thought presumed that rapidly proliferating cancer cells possess an acidic cytosol because of the Warburg effect and production of lactic acid generated by aerobic glycolysis. More recent work reveals a reversal of the pH gradient in many cancer cells because of upregulated efflux of lactate and protons across the plasma membrane, however, resulting in a slightly alkaline pHi (∼7.3–7.6) and a slightly acidic pHe (∼6.8–7.0), the reverse of normal cells (42, 43, 44, 45, 46). These changes in pHi and pHe because of altered proton (H^+^) flux drive diverse oncogenic cell behaviors, including cell proliferation, evasion from apoptosis, and cell migration through pathways and mechanisms that remain incompletely defined (42, 43, 44, 45, 46).

This study demonstrates that an alkaline pHi mimics growth factor–PI3K signaling to mTORC1 and mTORC2, pathways hyperactivated in many human tumors that contribute to their aberrant growth, proliferation, and survival (4, 71). We found that an elevated pHi is sufficient to increase mTORC1 and mTORC2 signaling in a PI3K-dependent manner in the absence of serum growth factors (Fig. 7*D*). Mechanistically, alkaline pHi increases PI3K activity and signaling to Akt, which leads to inhibitory phosphorylation of TSC2 and PRAS40 and dissociation of TSC from lysosomal membranes. These events enable Rheb to activate mTORC1 during growth factor limitation. Activation of PI3K by pHi explains why the activation of mTORC2 by alkaline pHi was only partially dependent on AMPK, as reported in our prior work (41). By mimicking PI3K action, alkaline pHi cooperates in parallel with amino acids to activate mTORC1 and cooperates in parallel with AMPK to activate mTORC2 (Fig. 7*D*). Functionally, alkaline pHi increases initiation of cap-dependent translation in a PI3K- and mTOR-dependent manner in the absence of growth factors through inhibitory phosphorylation of 4EBP1, thus enabling assembly of the eIF4F translation preinitiation complex (Fig. 7*D*). Alkaline pHi also protects against apoptosis caused by the withdrawal of growth factors in a PI3K- and mTOR-dependent manner.

In addition to finding that an alkaline pHi increases mTORC1 signaling, we found that an acidic pHi suppresses mTORC1 signaling, which agrees with the work of other groups (72, 73, 74). Balgi *et al.* (72) found that acidic pHi reduces mTORC1 signaling in a TSC-dependent manner, resulting in reduced protein synthesis. Walton *et al.* (73) found that acidic pHi induces a lysosomal pool of mTORC1 to move to a more peripheral subcellular localization, away from Rheb, thus limiting mTORC1 activity. Erra Diaz *et al.* (74) found that acidic pHi reduces mTORC1 signaling to direct the differentiation of dendritic cells into monocytes rather than macrophages. Physiologically, high-intensity exercise leads to rapid acidification of the cytosol in skeletal muscle, whereas pathologically, hypoxia/ischemia acidifies the cytosol. Downregulated mTORC1 signaling in both instances would ensure that cells within affected tissues do not engage in energetically costly anabolic metabolism.

Cancer cells often bear an alkaline pHi through the upregulated expression of transporters that actively extrude cytosolic acid (42, 43, 44, 45). A recent study demonstrated that increased expression of the sodium–proton antiporter NHEI or the lactate–proton symporter MCT4, both of which extrude H^+^ from the cytosol, increased the pHi of hematopoietic cells, which promoted their growth and proliferation in culture and *in vivo* (46). We speculate that elevated mTORC1 and/or mTORC2 signaling may have contributed to the hyperproliferative phenotype of the hematopoietic cells in this study, as we found that inhibition of NHEI with cariporide reduces mTORC1 signaling (this study) and mTORC2 signaling (our prior work) (41). Interestingly, Man *et al*. (46) found that elevated pHi mediated by overexpression of H^+^ transporters also increased the activity of several metabolic gatekeeper enzymes (*e.g.*, hexokinase 1, pyruvate kinase M2, glucose 6 phosphate dehydrogenase) that promote glycolytic and pentose phosphate carbon flux. Thus, an alkaline pHi appears to influence metabolic adaptation as well as classical oncogenic cell behaviors.

Cancer cells also upregulate glutaminolysis as part of a metabolic reprogramming response that not only provides fuel for growth and proliferation but also liberates ammonium (NH_4_^+^) as a waste byproduct. Merhi *et al.* (75) found that delivering ammonium to cells as either NH_4_Cl or NH_4_OH increased mTORC1 signaling weakly (4EBP1 P-T37/46) but increased PI3K (PRAS40 P-T246) and mTORC2 (Akt P-S473) signaling strongly. In addition, NH_4_Cl increased the proliferation of cells deprived of glutamine (75). Interestingly, we used NH_4_Cl as an alternative method of increasing cytosolic pH here and in our prior work (41). Similar to Merhi *et al.* (75), we found that NH_4_Cl increases PI3K and mTORC2 signaling strongly but mTORC1 signaling modestly, likely by increasing pHi.

Evasion of apoptosis represents an established hallmark of cancer cells (76). We found that increased pHi attenuated the induction of apoptosis caused by growth factor withdrawal. The ability of alkaline pHi to protect against apoptosis was blunted in a statistically significant manner in cells treated with the PI3K inhibitor BYL719 and the mTOR inhibitor Torin1. These data suggest that an alkaline pHi in cancer cells may contribute to tumorigenesis through increased cell survival mediated by PI3K–mTOR signaling. While the allosteric mTORC1-specific inhibitor rapamycin blunted the evasion of apoptosis caused by alkaline pHi modestly during growth factor withdrawal, the effect was not statistically significant. In Rictor knockout MEFs lacking mTORC2 function, however, alkaline pHi and low CO_2_ (*i.e.*, 0.1%) increased S6K1 phosphorylation and attenuated apoptosis concomitantly in a rapamycin-sensitive manner relative to physiological pH and normal CO_2_ (*i.e.*, 7.5%) during growth factor withdrawal. It is important to note that as some mTORC1 substrates display only partial sensitivity to rapamycin (*e.g.*, 4EBP1) (77, 78), a rapamycin-insensitive mTORC1 substrate may contribute to the attenuation of apoptosis. While limited reports have documented a role for mTORC1 in promoting cell survival, knockout of TSC2 and activation of mTORC1 in a mouse model of lymphoma accelerated oncogenesis, suppressed tumor apoptosis, and decreased animal survival *in vivo* (79). Treatment of these mice with rapamycin increased apoptosis in the lymphomas and prolonged animal survival (79), suggesting that mTORC1 may promote tumor cell survival in certain oncogenic contexts. Taken together, our results suggest that while mTORC1 may contribute to the evasion of apoptosis by alkaline pHi during growth factor limitation, mTORC2 likely represents the major mTOR complex that suppresses apoptosis in response to alkaline pHi.

It will be important in the future to determine how an alkaline pHi increases PI3K activity, leading to elevated mTORC1 and mTORC2 signaling. Recent research indicates that changes in pHi alter the structure and function of pH-sensitive proteins (aka, pH sensors) through dynamic protonation and deprotonation, a post-translational modification akin to phosphorylation, ubiquitination, acetylation, and so forth (45). In fact, recurring charge-changing mutations (*e.g.*, Arg to His) have been documented in the epidermal growth factor receptor, the tumor suppressor protein p53, Ras-GRP1, and β-catenin, which alters their activities in cancer cells bearing an alkaline pHi and contributes to the transformed behaviors of the cells bearing these mutations (44, 80, 81, 82). Theoretically, altered protonation of PI3K subunits, either the catalytic p110 subunit or the regulatory p85 subunit, could increase PI3K activity. In addition, certain recurring mutations in PI3K subunits that drive oncogenesis could render PI3K activity more sensitive to the increased pHi of cancer cells, such as occurs in documented pH sensors (*e.g.*, epidermal growth factor receptor, p53, β-catenin) bearing charge-changing Arg to His mutations (44, 80, 81). Altered protonation mediated by alkaline pHi may also act upstream of PI3K, possibly at the level of the lipid phosphatase PTEN, growth factor receptors, or their adaptors.

Collectively, these findings reveal that pHi functions as an internal rheostat that fine-tunes the level of PI3K–mTOR signaling, likely in response to physiological and pathological cues. In the context of cancer, we propose that elevated PI3K–mTORC1 and/or PI3K–mTORC2 signaling may contribute to the growth, proliferation, and survival of cancer cells bearing an alkaline cytosolic pH in growing tumors deprived of essential growth factors, prior to the onset of angiogenesis. As a final note, this study provides a cautionary tale highlighting the importance of controlling pH appropriately in cell cultures, as inadvertent alteration of the pHe affects pHi, cell signaling, and cell function (48).

## Experimental procedures

### Materials

General chemicals were from ThermoFisher Scientific or MilliporeSigma. Protein A-Sepharose beads were from GE Healthcare (catalog no.: GE17-0780-01). NP40, Brij35, and CHAPS detergents were from Pierce. Complete Protease Inhibitor Cocktail (EDTA-free) tablets was from MilliporeSigma (catalog no.: 11836170001). 7-methyl-GTP-Sepharose 4B beads were from GE Healthcare (catalog no.: 275025). Immobilon-P polyvinylidene difluoride (PVDF) membrane (0.45 μM) was from MilliporeSigma. Reagents for enhanced chemiluminescence were from either Alkali Scientific (Bright Star; catalog no.: XR92) or Advansta (WesternBright Sirius horseradish peroxidase [HRP] substrate). Torin1 was a gift from Dr David Sabatini. Rapamycin was from Calbiochem (catalog no.: 553210). BYL719 was from Selleck (catalog no.: 1020). MK2206 was from Selleck (catalog no.: 1078). NH_4_Cl was from ThermoFisher (catalog no.: A661). cSNARF1-AM was from Invitrogen (catalog no.: C1272). Cariporide was from MilliporeSigma (catalog no.: SML1360).

### Antibodies

The following antibodies were from Cell Signaling Technology: Akt P-S473 (catalog no.: 4060), Akt P-T308 (catalog no.: 4056), Akt P-T450 (catalog no.: 9267), Akt (catalog no.: 9272), mTOR (catalog no.: 2972), Raptor (catalog no.: 2280), S6K1 P-T389 (catalog no.: 9234), 4EBP1 P-S65 (catalog no.: 9451), 4EBP1 P-T70 (catalog no.: 9455), 4EBP1 (catalog no.: 9452), eIF4E (catalog no.: 9742), eIF4G (catalog no.: 2498), PRAS40 P-S246 (catalog no.: 2691), PRAS40 (catalog no.: 2997), TSC2 P-S939 (catalog no.: 3615), TSC2 (catalog no.: 4308), ULK1 P-S757 (catalog no.: 14202), ULK1 (catalog no.:8054), cCasp3 (catalog no.: 9664), cleaved PARP (catalog no.: 9544), and α-tubulin (catalog no.: 2144). mTOR P-S2481 was from MilliporeSigma (catalog no.: 09-343). The antipuromycin antibody was from MilliporeSigma (catalog no.: MABE343). The S6K1 P-T229 antibody was from Abcam (catalog no.: ab59208). Antipeptide polyclonal antibodies generated against Rictor (amino acids 6–20; human) and S6K1 (amino acids 485–502; rat 70 kDa isoform) were custom-made with the aid of Covance, as described (83). Donkey anti-rabbit-HRP secondary antibodies for Western blotting were from Jackson ImmunoResearch (catalog no.: 711-095-152) as were Alexa 488-conjugated anti-rabbit-HRP (catalog no.: 711545152) and Alexa 594-conjugated rat-HRP (catalog no.: 112585167) antibodies for immunofluorescence microscopy.

### Cell culture and treatments

Cell lines (MEFs, C2C12, U2OS, HEK293, HeLa, and MCF7) were cultured in DMEM containing high glucose (4.5 g/l), glutamine (584 mg/l), and sodium pyruvate (110 mg/l) (Life Technologies; catalog no.: 11995-065) supplemented with 10% FBS (Life Technologies; catalog no.: 10347-028) and incubated at 37 °C in a humidified atmosphere with 7.5% CO_2_. To serum-starve cells, cells were washed once in DMEM containing 20 mM Hepes (pH 7.2) and then refed with this medium overnight (∼16 h). To amino acid–starve cells, cells were washed once in PBS containing Ca^2+^ and Mg^+^ and then refed with amino acid-free DMEM (US Biologicals; catalog no.: D9800-13) lacking or containing dialyzed FBS (10%) (Life Technologies; catalog no.: A33820-01) for 50 min. After amino acid deprivation, cells were stimulated with amino acids by adding an amino acid solution (RPMI1640 Amino Acid Solution [50×]; Sigma; catalog no.: R7131) supplemented with l-glutamine (Sigma; catalog no.: G8540) to a final concentration of (1×) (roughly the concentration of amino acids found in RPMI media). The pH of the commercial amino acid solution (∼ pH 10) was adjusted to physiological pH 7.4 with 1 N HCl prior to addition to cells. To culture cells at an alkaline pHe, and thus to raise pHi, the pH of the DMEM was adjusted to pH 8.3 (or the pH indicated) using NaOH. To maintain DMEM at an alkaline pH (*e.g.*, pH 8.3) for a longer period, cells cultured in DMEM (pH 8.3) were incubated at 37 °C in a humidified atmosphere set to low CO_2_ (0.1%). Where indicated, cells were pretreated with various drugs or chemicals for 30 min: Torin1 (100 nM), BYL719 (10 μM), MK2206 (10 μM), cariporide (10 μM), and NH_4_Cl (10 mM).

### Cell lysis, immunoprecipitation, and immunoblotting

Unless indicated otherwise, cells were washed twice with ice-cold PBS, lysed in ice-cold buffer containing NP-40 (0.5%) and Brij35 (0.1%), and incubated on ice (15 min), as described (83). To maintain the detergent-sensitive mTOR–Raptor interaction, cells were lysed in ice-cold buffer containing CHAPS (0.3%) detergent. Lysates were clarified by spinning at 13,200 rpm for 5 min at 4 °C, and the postnuclear supernatants were collected. Bradford assay was used to normalize protein levels for immunoprecipitation and immunoblot analyses. For immunoprecipitation, whole-cell lysates were incubated with antibodies for 2 h at 4 °C, followed by incubation with Protein A-Sepharose beads for 1 h. For 7-methyl-GTP Sepharose pull-down assays, cells lysed in ice-cold buffer containing NP-40 (0.5%) and Brij35 (0.1%) were incubated with 7-methyl-GTP Sepharose beads for 2 h. Sepharose beads were washed three times in lysis buffer and resuspended in 1× sample buffer. Samples were resolved on SDS-PAGE gels and transferred to PVDF membranes in Towbin transfer buffer containing 0.02% SDS, as described (83). Immunoblotting was performed by blocking PVDF membranes in Tris-buffered saline (TBS) pH 7.5 with 0.1% Tween-20 (TBST) containing 3% nonfat dry milk, as described (83) and incubating the membranes in TBST containing 2% bovine serum albumin containing primary antibodies or secondary HRP-conjugated antibodies. Blots were developed by enhanced chemiluminescence and detected digitally with a UVP ChemStudio Imaging System from Analytik Jena.

### Isolation of primary MEFs

Primary MEFs were isolated from day 12.5 mouse embryos, as described (84, 85).

### Lentiviral transduction

*Rictor*^*−/−*^ MEFs stably expressing vector control (pPPM) or HA-Rictor were generated by lentiviral transduction. The HA-tagged Rictor complementary DNA was subcloned into a modified lentiviral vector, pHAGE-Puro-MCS (pPPM) (86) (modified by Amy Hudson; Medical College of Wisconsin). Lentivirus particles were packaged in HEK293T cells by cotransfection with empty vector or pPPM/HA-Rictor together with pRC/Tat, pRC/Rev, pRC/gag-pol, and pMD/VSV-G plasmids using Mirus TransIT-LT1 transfection reagent. Supernatants containing viral particles were collected 48 h post-transfection and filtered through a 0.45 μm filter. *Rictor*^*−/−*^ MEFs were infected with fresh viral supernatants containing 8 μg/ml polybrene. About 24 h postinfection, cells were selected in DMEM/10% FBS supplemented with 3 μg/ml puromycin.

### Quantification of PIP_3_ to measure PI3K activity

Levels of PIP_3_ were measured using an ELISA-based kit according to the manufacturer’s instructions (Echelon; catalog no.: K2500s). MEFs were cultured on 15 cm plates and treated with media at pH 7.4 or 8.3, drugs, and/or insulin. The media were aspirated, 10 ml of ice-cold 0.5 M TCA was added, and the plate was incubated on ice for 5 min. Cells were scraped and collected into a 15 ml tube on ice and centrifuged at 950*g* for 7 min at 4 °C. The pellet was resuspended in 3 ml of 5% tricarboxylic acid/1 mM EDTA, vortexed, centrifuged at 3000 rpm for 5 min, and washed once again in 3 ml 5% trichloroacetic acid/1 mM EDTA. Neutral lipids were next extracted by adding 3 ml of MeOH:CHCl_3_ (2:1) to the pellet and vortexing 10 min followed by centrifugation at 950*g* for 5 min. The supernatant was discarded, and this extraction step was repeated once more. To extract acidic lipids, 2.25 ml of MeOH:CHCl3:HCl (12 N) (80:40:1) was added, and the solution was vortexed continuously for 25 min. Extracts were centrifuged at 950*g* for 5 min, and the supernatant was transferred to a new 15 ml tube. About 0.75 ml of CHCl_3_ and 1.35 ml of 0.1 M HCl was added to the supernatant, vortexed, and centrifuged at 950*g* for 5 min to separate the lower organic and upper aqueous phases. The organic phase (∼1.45 ml) was transferred to a new tube for the measurement of PIP_3_. All samples were dried in a vacuum dryer for 1 h. PIP_3_ samples were resuspended in 140 ml of PBS–Tween +3% Protein Stabilizer (provided in the Echelon kit). Samples were sonicated in an ice-water bath for 10 min and spun down. The experiment was performed four times with technical duplicates. PIP_3_ levels were measured by ELISA, according to the manufacturer’s instructions (Echelon; catalog no.: K2500s).

### cSNARF-1AM microscopy

MEFs were cultured on 8-well cover glass bottom chambers (Lab-Tek; catalog no.: 155409) and loaded with cSNARF1-AM (10 μM) (Thermo Fisher; catalog no.: C1272) for 30 min at 37 °C in serum-free DMEM (pH 7.4). The cells were then rinsed with serum-free DMEM (pH 7.4) and refed with this medium. Live-cell imaging (0–10 min) of cells cultured in serum-free DMEM (pH 7.4 or 8.3) was performed using a Nikon A1 confocal microscope equipped with a stage-top incubator that maintains temperature (37 °C) and CO_2_ (7.5%). Images were acquired using a 40× oil objective (1.3 numerical aperture) at a resolution of 1024 × 1024 and an optical thickness of 1.18 μM (confocal aperture set at three airy units). The cSNARF1-AM signal was excited with an argon laser at 488 nm, and images sets were collected simultaneously in two emission bandpass filters at 553 to 618 nm (for 580 nm) and 663 to 738 nm (for 640 nm). Nikon Elements software was used to create and pseudo-color the 640:580 nm ratiometric images with values ranging from 0 (*violet*) to 2.0 (*red*). MetaMorph software (from MDS Analytical Technologies) was used to quantify the ratiometric images by measuring the average ratio of a region of interest within one cell, and 25 cells per image were quantified. A change in the 640:580 nm ratio and accompanying pseudo-colored image reflects a change in pHi.

### Confocal immunofluorescence microscopy

MEFs were seeded on glass coverslips in 6-well plates and serum-starved overnight (∼16 h). Following various treatments, cells were washed with PBS containing Ca^2+^ and Mg^2+^ (aka, PBS^+^) and fixed with 3.7% formaldehyde for 10 min. Cells were then washed twice with PBS^+^, permeabilized in 0.2% TX-100 for 5 min, washed twice with PBS^+^, and blocked in 0.2% fish skin gelatin (FSG) for 1 h. Cover slips were inverted on primary antibodies (1:100 dilution) (rabbit anti-TSC2; catalog no.: CST4308; rat anti-LAMP1; catalog no.: ab25245) diluted in PBS^+^ containing 0.2% FSG and incubated overnight. Cells were washed three times with PBS^+^, and cover slips were inverted on Alexa 488-conjugated anti-rabbit antibody (1:500 dilution) (Jackson; catalog no.: 711545152) or Alexa 594-conjugated antirat antibody (1:500 dilution) (Jackson; catalog no.: 112585167) diluted in PBS^+^/0.2% FSG, incubated for 1 h, and washed three times with PBS^+^. Cover slips were mounted on a slide using Prolong Gold with 4′,6-diamidino-2-phenylindole (Invitrogen). Images were captured using a 40× objective lens on a Zeiss LSM800 Confocal Laser Scanning Microscope, and images were analyzed using Fiji.

### Measurement of protein synthesis

Protein synthesis was measured by SUnSET assay, as described (87). Briefly, MEFs seeded to 6-well plates were serum-starved overnight (∼16 h) and refed with serum-free DMEM at pH 7.4 or 8.3 for 60 min. To maintain DMEM at an alkaline pH (*i.e.*, pH 8.3) for a longer period, the cells were incubated at 37 °C in a humidified atmosphere at low CO_2_ (*i.e.*, 0.1% CO_2_). Puromycin (10 μg/ml) was added for the last 10 min of refeeding. Cells were lysed with ice-cold lysis buffer containing NP-40 (0.5%) and Brij35 (0.1%), and whole-cell lysates were immunoblotted with an antipuromycin antibody. Puromycin incorporation was analyzed using ImageJ (aka, Fiji) software. Equal protein loading of SDS-PAGE gels was confirmed by staining with SimplyBlue SafeStain (Invitrogen; catalog no.: LC6060), according to the manufacturer’s instructions.

### Image editing

Adobe Photoshop was used for image preparation, using only the levels, brightness, and contrast controllers equivalently over the entire image.

### Statistical analysis

Results are presented as mean ± SD. Data were analyzed by one-way ANOVA followed by Tukey's multiple comparisons test. Values of *p* < 0.05 were considered significant. ∗*p* < 0.05; ∗∗*p* < 0.01; ∗∗∗*p* < 0.001; ∗∗∗∗*p* < 0.00001.

## Data availability

The data shown in Figure 1, Figure 2, Figure 3, Figure 4, Figure 5, Figure 6, Figure 7 are available upon request. To request these data, please contact Diane C. Fingar, PhD (dfingar@umich.edu) and/or Dubek Kazyken, PhD (dkazyken@umich.edu). This article also contains supporting information, specifically Figs. S1 and S2 and legends.

## Supporting information

This article contains supporting information.

## Conflict of interest

The authors declare that they have no conflicts of interest with the contents of this article.

## References

[1] Ben-Sahra I., Manning B.D. (2017). mTORC1 signaling and the metabolic control of cell growth. Curr. Opin. Cell Biol..

[2] Kim J., Guan K.L. (2019). mTOR as a central hub of nutrient signalling and cell growth. Nat. Cell Biol..

[3] Fu W., Hall M.N. (2020). Regulation of mTORC2 signaling. Genes (Basel).

[4] Liu G.Y., Sabatini D.M. (2020). mTOR at the nexus of nutrition, growth, ageing and disease. Nat. Rev. Mol. Cell Biol..

[5] Szwed A., Kim E., Jacinto E. (2021). Regulation and metabolic functions of mTORC1 and mTORC2. Physiol. Rev..

[6] Battaglioni S., Benjamin D., Walchli M., Maier T., Hall M.N. (2022). mTOR substrate phosphorylation in growth control. Cell.

[7] Kim D.H., Sarbassov D.D., Ali S.M., King J.E., Latek R.R., Erdjument-Bromage H. (2002). mTOR interacts with raptor to form a nutrient-sensitive complex that signals to the cell growth machinery. Cell.

[8] Hara K., Maruki Y., Long X., Yoshino K., Oshiro N., Hidayat S. (2002). Raptor, a binding partner of target of rapamycin (TOR), mediates TOR action. Cell.

[9] Sarbassov D.D., Ali S.M., Kim D.H., Guertin D.A., Latek R.R., Erdjument-Bromage H. (2004). Rictor, a novel binding partner of mTOR, defines a rapamycin-insensitive and raptor-independent pathway that regulates the cytoskeleton. Curr. Biol..

[10] Jacinto E., Loewith R., Schmidt A., Lin S., Ruegg M.A., Hall A. (2004). Mammalian TOR complex 2 controls the actin cytoskeleton and is rapamycin insensitive. Nat. Cell Biol..

[11] Manning B.D., Toker A. (2017). AKT/PKB signaling. Navigating Netw. Cell.

[12] Hoxhaj G., Manning B.D. (2020). The PI3K-AKT network at the interface of oncogenic signalling and cancer metabolism. Nat. Rev. Cancer.

[13] Inoki K., Li Y., Zhu T., Wu J., Guan K.L. (2002). TSC2 is phosphorylated and inhibited by Akt and suppresses mTOR signalling. Nat. Cell Biol..

[14] Manning B.D., Tee A.R., Logsdon M.N., Blenis J., Cantley L.C. (2002). Identification of the tuberous sclerosis complex-2 tumor suppressor gene product tuberin as a target of the phosphoinositide 3-kinase/akt pathway. Mol. Cell.

[15] Menon S., Dibble C.C., Talbott G., Hoxhaj G., Valvezan A.J., Takahashi H. (2014). Spatial control of the TSC complex integrates insulin and nutrient regulation of mTORC1 at the lysosome. Cell.

[16] Inoki K., Li Y., Xu T., Guan K.L. (2003). Rheb GTPase is a direct target of TSC2 GAP activity and regulates mTOR signaling. Genes Dev..

[17] Tee A.R., Manning B.D., Roux P.P., Cantley L.C., Blenis J. (2003). Tuberous sclerosis complex gene products, Tuberin and Hamartin, control mTOR signaling by acting as a GTPase-activating protein complex toward Rheb. Curr. Biol..

[18] Garami A., Zwartkruis F.J., Nobukuni T., Joaquin M., Roccio M., Stocker H. (2003). Insulin activation of Rheb, a mediator of mTOR/S6K/4E-BP signaling, is inhibited by TSC1 and 2. Mol. Cell.

[19] Long X., Ortiz-Vega S., Lin Y., Avruch J. (2005). Rheb binding to mammalian target of rapamycin (mTOR) is regulated by amino acid sufficiency. J. Biol. Chem..

[20] Long X., Lin Y., Ortiz-Vega S., Yonezawa K., Avruch J. (2005). Rheb binds and regulates the mTOR kinase. Curr. Biol..

[21] Yang H., Jiang X., Li B., Yang H.J., Miller M., Yang A. (2017). Mechanisms of mTORC1 activation by RHEB and inhibition by PRAS40. Nature.

[22] Liu P., Gan W., Chin Y.R., Ogura K., Guo J., Zhang J. (2015). PtdIns(3,4,5)P3-dependent activation Mtorc2 kinase complex. Cancer Discov..

[23] Sarbassov D.D., Guertin D.A., Ali S.M., Sabatini D.M. (2005). Phosphorylation and regulation of Akt/PKB by the rictor-mTOR complex. Science.

[24] Alessi D.R., Andjelkovic M., Caudwell B., Cron P., Morrice N., Cohen P. (1996). Mechanism of activation of protein kinase B by insulin and IGF-1. EMBO J..

[25] Jacinto E., Facchinetti V., Liu D., Soto N., Wei S., Jung S.Y. (2006). SIN1/MIP1 maintains rictor-mTOR complex integrity and regulates Akt phosphorylation and substrate specificity. Cell.

[26] Guertin D.A., Stevens D.M., Thoreen C.C., Burds A.A., Kalaany N.Y., Moffat J. (2006). Ablation in mice of the mTORC components raptor, rictor, or mLST8 reveals that mTORC2 is required for signaling to Akt-FOXO and PKCalpha, but not S6K1. Dev. Cell.

[27] Sancak Y., Peterson T.R., Shaul Y.D., Lindquist R.A., Thoreen C.C., Bar-Peled L. (2008). The Rag GTPases bind raptor and mediate amino acid signaling to mTORC1. Science.

[28] Sancak Y., Bar-Peled L., Zoncu R., Markhard A.L., Nada S., Sabatini D.M. (2010). Ragulator-rag complex targets mTORC1 to the lysosomal surface and is necessary for its activation by amino acids. Cell.

[29] Demetriades C., Doumpas N., Teleman A.A. (2014). Regulation of TORC1 in response to amino acid starvation *via* lysosomal recruitment of TSC2. Cell.

[30] Demetriades C., Plescher M., Teleman A.A. (2016). Lysosomal recruitment of TSC2 is a universal response to cellular stress. Nat. Commun..

[31] Yang G., Humphrey S.J., Murashige D.S., Francis D., Wang Q.P., Cooke K.C. (2019). RagC phosphorylation autoregulates mTOR complex 1. EMBO J..

[32] Yao Y., Hong S., Ikeda T., Mori H., MacDougald O.A., Nada S. (2020). Amino acids enhance polyubiquitination of Rheb and its binding to mTORC1 by blocking lysosomal ATXN3 deubiquitinase activity. Mol. Cell.

[33] Ma X.M., Blenis J. (2009). Molecular mechanisms of mTOR-mediated translational control. Nat. Rev. Mol. Cell Biol..

[34] Roux P.P., Topisirovic I. (2018). Signaling pathways involved in the regulation of mRNA translation. Mol. Cell Biol..

[35] Gingras A.C., Raught B., Gygi S.P., Niedzwiecka A., Miron M., Burley S.K. (2001). Hierarchical phosphorylation of the translation inhibitor 4E-BP1. Genes Dev..

[36] Kazyken D., Magnuson B., Bodur C., Acosta-Jaquez H.A., Zhang D., Tong X. (2019). AMPK directly activates mTORC2 to promote cell survival during acute energetic stress. Sci. Signal..

[37] Jeon S.M., Hay N. (2012). The dark face of AMPK as an essential tumor promoter. Cell Logist..

[38] Svensson R.U., Shaw R.J. (2012). Cancer metabolism: tumour friend or foe. Nature.

[39] Hardie D.G. (2013). The LKB1-AMPK pathway-friend or foe in cancer?. Cancer cell.

[40] Hardie D.G., Alessi D.R. (2013). LKB1 and AMPK and the cancer-metabolism link - ten years after. BMC Biol..

[41] Kazyken D., Lentz S.I., Fingar D.C. (2021). Alkaline intracellular pH (pHi) activates AMPK-mTORC2 signaling to promote cell survival during growth factor limitation. J. Biol. Chem..

[42] Reshkin S.J., Greco M.R., Cardone R.A. (2014). Role of pHi, and proton transporters in oncogene-driven neoplastic transformation. Philos. Trans. R. Soc. Lond. B Biol. Sci..

[43] White K.A., Grillo-Hill B.K., Barber D.L. (2017). Cancer cell behaviors mediated by dysregulated pH dynamics at a glance. J. Cell Sci..

[44] White K.A., Kisor K., Barber D.L. (2019). Intracellular pH dynamics and charge-changing somatic mutations in cancer. Cancer Metast. Rev..

[45] Liu Y., White K.A., Barber D.L. (2020). Intracellular pH regulates cancer and stem cell behaviors: a protein dynamics perspective. Front. Oncol..

[46] Man C.H., Mercier F.E., Liu N., Dong W., Stephanopoulos G., Jiang L. (2022). Proton export alkalinizes intracellular pH and reprograms carbon metabolism to drive normal and malignant cell growth. Blood.

[47] Lamming D.W., Ye L., Katajisto P., Goncalves M.D., Saitoh M., Stevens D.M. (2012). Rapamycin-induced insulin resistance is mediated by mTORC2 loss and uncoupled from longevity. Science.

[48] Michl J., Park K.C., Swietach P. (2019). Evidence-based guidelines for controlling pH in mammalian live-cell culture systems. Commun. Biol..

[49] Perdikis D.A., Davies R., Zhuravkov A., Brenner B., Etter L., andBasson M.D. (1998). Differential effects of mucosal pH on human (Caco-2) intestinal epithelial cell motility, proliferation, and differentiation. Dig. Dis. Sci..

[50] Seglen P.O., Grinde B., Solheim A.E. (1979). Inhibition of the lysosomal pathway of protein degradation in isolated rat hepatocytes by ammonia, methylamine, chloroquine leupeptin. Eur. J. Biochem..

[51] Roos A., andBoron W.F. (1981). Intracellular pH. Physiol. Rev..

[52] Di Sario A., Bendia E., Omenetti A., De Minicis S., Marzioni M., Kleemann H.W. (2007). Selective inhibition of ion transport mechanisms regulating intracellular pH reduces proliferation and induces apoptosis in cholangiocarcinoma cells. Dig Liver Dis..

[53] Persi E., Duran-Frigola M., Damaghi M., Roush W.R., Aloy P., Cleveland J.L. (2018). Systems analysis of intracellular pH vulnerabilities for cancer therapy. Nat. Commun..

[54] Harguindey S., Arranz J.L., Polo Orozco J.D., Rauch C., Fais S., Cardone R.A. (2013). Cariporide and other new and powerful NHE1 inhibitors as potentially selective anticancer drugs--an integral molecular/biochemical/metabolic/clinical approach after one hundred years of cancer research. J. Transl. Med..

[55] Moore R.D. (1979). Elevation of intracellular pH by insulin in frog skeletal muscle. Biochem. Biophys. Res. Commun..

[56] Moore R.D. (1981). Stimulation of Na:H exchange by insulin. Biophys. J..

[57] Fidelman M.L., Seeholzer S.H., Walsh K.B., Moore R.D. (1982). Intracellular pH mediates action of insulin on glycolysis in frog skeletal muscle. Am. J. Physiol..

[58] L'Allemain G., Paris S., Pouyssegur J. (1984). Growth factor action and intracellular pH regulation in fibroblasts. Evidence for a major role of the Na+/H+ antiport. J. Biol. Chem..

[59] Paris S., Pouyssegur J. (1984). Growth factors activate the Na+/H+ antiporter in quiescent fibroblasts by increasing its affinity for intracellular H+. J. Biol. Chem..

[60] Ives H.E., Daniel T.O. (1987). Interrelationship between growth factor-induced pH changes and intracellular Ca2+. Proc. Natl. Acad. Sci. U. S. A..

[61] Pouyssegur J., Franchi A., L'Allemain G., Paris S. (1985). Cytoplasmic pH, a key determinant of growth factor-induced DNA synthesis in quiescent fibroblasts. FEBS Lett..

[62] Harrington L.S., Findlay G.M., Gray A., Tolkacheva T., Wigfield S., Rebholz H. (2004). The TSC1-2 tumor suppressor controls insulin-PI3K signaling *via* regulation of IRS proteins. J. Cell Biol..

[63] Harrington L.S., Findlay G.M., Lamb R.F. (2005). Restraining PI3K: mTOR signalling goes back to the membrane Trends. Biochem. Sci..

[64] Shah O.J., Wang Z., Hunter T. (2004). Inappropriate activation of the TSC/Rheb/mTOR/S6K cassette induces IRS1/2 depletion, insulin resistance, and cell survival deficiencies. Curr. Biol..

[65] Tato I., Bartrons R., Ventura F., Rosa J.L. (2011). Amino acids activate mammalian target of rapamycin complex 2 (mTORC2) *via* PI3K/Akt signaling. J. Biol. Chem..

[66] Dalle Pezze P., Ruf S., Sonntag A.G., Langelaar-Makkinje M., Hall P., Heberle A.M. (2016). A systems study reveals concurrent activation of AMPK and mTOR by amino acids. Nat. Commun..

[67] Facchinetti V., Ouyang W., Wei H., Soto N., Lazorchak A., Gould C. (2008). The mammalian target of rapamycin complex 2 controls folding and stability of Akt and protein kinase C. EMBO J..

[68] Oh W.J., Wu C.C., Kim S.J., Facchinetti V., Julien L.A., Finlan M. (2010). mTORC2 can associate with ribosomes to promote cotranslational phosphorylation and stability of nascent Akt polypeptide. EMBO J..

[69] Soliman G.A., Acosta-Jaquez H.A., Dunlop E.A., Ekim B., Maj N.E., Tee A.R. (2010). mTOR Ser-2481 autophosphorylation monitors mTORC-specific catalytic activity and clarifies rapamycin mechanism of action. J. Biol. Chem..

[70] Ravi V., Jain A., Mishra S., Sundaresan N.R. (2020). Measuring protein synthesis in cultured cells and mouse tissues using the non-radioactive SUnSET assay. Curr. Protoc. Mol. Biol..

[71] Menon S., Manning B.D. (2008). Common corruption of the mTOR signaling network in human tumors. Oncogene.

[72] Balgi A.D., Diering G.H., Donohue E., Lam K.K., Fonseca B.D., Zimmerman C. (2011). Regulation of mTORC1 signaling by pH. PLoS One.

[73] Walton Z.E., Patel C.H., Brooks R.C., Yu Y., Ibrahim-Hashim A., Riddle M. (2018). Acid suspends the circadian clock in hypoxia through inhibition of mTOR. Cell.

[74] Erra Diaz F., Ochoa V., Merlotti A., Dantas E., Mazzitelli I., Gonzalez Polo V. (2020). Extracellular acidosis and mTOR inhibition drive the differentiation of human monocyte-derived dendritic cells. Cell Rep..

[75] Merhi A., Delree P., Marini A.M. (2017). The metabolic waste ammonium regulates mTORC2 and mTORC1 signaling. Sci. Rep..

[76] Hanahan D., Weinberg R.A. (2000). The hallmarks of cancer. Cell.

[77] Choo A.Y., Blenis J. (2009). Not all substrates are treated equally: implications for mTOR, rapamycin-resistance cancer theraphy. Cell Cycle.

[78] Choo A.Y., Yoon S.O., Kim S.G., Roux P.P., Blenis J. (2008). Rapamycin differentially inhibits S6Ks and 4E-BP1 to mediate cell-type-specific repression of mRNA translation. Proc. Natl. Acad. Sci. U. S. A..

[79] Mills J.R., Hippo Y., Robert F., Chen S.M., Malina A., Lin C.J. (2008). mTORC1 promotes survival through translational control of Mcl-1. Proc. Natl. Acad. Sci. U. S. A..

[80] Vercoulen Y., Kondo Y., Iwig J.S., Janssen A.B., White K.A., Amini M. (2017). A Histidine pH sensor regulates activation of the Ras-specific guanine nucleotide exchange factor RasGRP1. Elife.

[81] White K.A., Grillo-Hill B.K., Esquivel M., Peralta J., Bui V.N., Chire I. (2018). beta-Catenin is a pH sensor with decreased stability at higher intracellular pH. J. Cell Biol..

[82] White K.A., Ruiz D.G., Szpiech Z.A., Strauli N.B., Hernandez R.D., Jacobson M.P. (2017). Cancer-associated arginine-to-histidine mutations confer a gain in pH sensing to mutant proteins. Sci. Signal..

[83] Acosta-Jaquez H.A., Keller J.A., Foster K.G., Ekim B., Soliman G.A., Feener E.P. (2009). Site-specific mTOR phosphorylation promotes mTORC1-mediated signaling and cell growth. Mol. Cell Biol..

[84] Todaro G.J., Green H. (1963). Quantitative studies of the growth of mouse embryo cells in culture and their development into established lines. J. Cell Biol..

[85] Bodur C., Kazyken D., Huang K., Tooley A.S., Cho K.W., Barnes T.M. (2022). TBK1-mTOR signaling attenuates obesity-linked hyperglycemia and insulin resistance. Diabetes.

[86] Mostoslavsky G., Fabian A.J., Rooney S., Alt F.W., Mulligan R.C. (2006). Complete correction of murine Artemis immunodeficiency by lentiviral vector-mediated gene transfer. Proc. Natl. Acad. Sci. U. S. A..

[87] Schmidt E.K., Clavarino G., Ceppi M., Pierre P. (2009). SUnSET, a nonradioactive method to monitor protein synthesis. Nat. Methods.

