# Supplementary material for: Alkaline intracellular pH (pHi) increases PI3K activity to promote mTORC1 and mTORC2 signaling and function during growth factor limitation
Source: J Biol Chem. 2023 Jul 26;299(9):105097. doi: 10.1016/j.jbc.2023.105097 (PMC10477693; doi:10.1016/j.jbc.2023.105097)
Supplement: Supporting information [file mmc1.pdf]

Supporting Information

Supplementary Figure S1

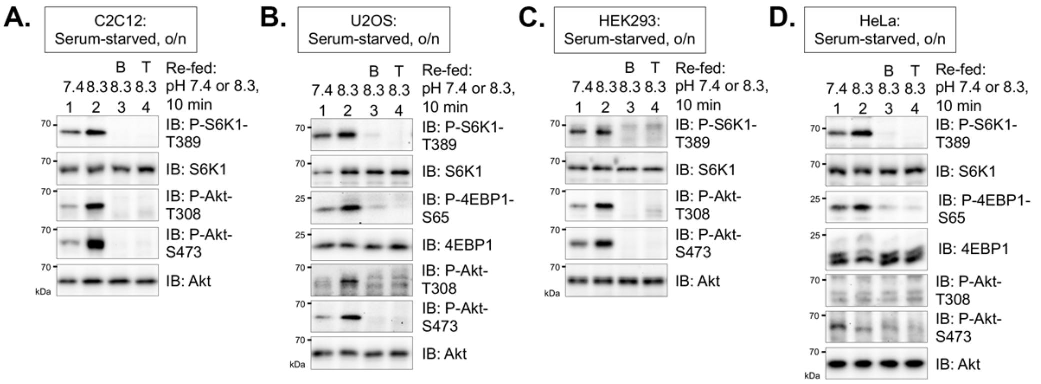

Figure S1 (related to Figure 1): **Alkaline extracellular pH (pHe) increases mTORC1 and mTORC2 signaling in the absence of serum growth factors in other cell lines**

A. C2C12 myoblasts were serum-starved overnight (o/n), pre-treated without or with BYL719 (BYL) [10 μM] or Torin1 (T) [100 nM] (30 min), and re-fed with serum-free DMEM at pH 7.4 or pH 8.3 (10 min) in the absence or presence of the drugs. Whole-cell lysates were immunoblotted as indicated.

B. U2OS osteosarcoma cells were treated as in **A**.

C. HEK293 cervical cancer cells were treated as in **A**.

D. HeLa cells were treated as in **A**.

Supplementary Figure S2

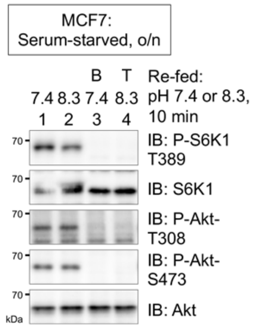

Figure S2 (related to Figure 4): **Alkaline pHi fails to increase mTORC1 and mTORC2 signaling in MCF7 breast cancer cells, which bear an oncogenic mutation in *PIK3CA*.**

MCF7 cells were serum-starved overnight (o/n), pre-treated without or with BYL719 (BYL) [10 μM] or Torin1 (T) [100 nM] (30 min), and re-fed with serum-free DMEM at pH 7.4 or pH 8.3 (10 min) in the absence or presence of the drugs. Whole-cell lysates were immunoblotted as indicated.
